# Involvement of metalloproteinase and nitric oxide synthase/nitric oxide mechanisms in early decidual angiogenesis–vascularization of normal and experimental pathological mouse placenta related to maternal alcohol exposure

**DOI:** 10.3389/fcell.2023.1207671

**Published:** 2023-08-16

**Authors:** Gisela Soledad Gualdoni, Camila Barril, Patricia Verónica Jacobo, Liliana Nazareth Pacheco Rodríguez, Elisa Cebral

**Affiliations:** Laboratorio de Reproducción y Fisiología Materno-Embrionaria, Instituto de Biodiversidad y Biología Experimental y Aplicada (IBBEA), Consejo Nacional de Investigaciones Científicas y Tecnológicas (CONICET), Departamento de Biodiversidad y Biología Experimental (DBBE), Facultad de Ciencias Exactas y Naturales, Universidad de Buenos Aires, Buenos Aires, Argentina

**Keywords:** placental vascularization, maternal angiogenesis, MMPs, NO, alcohol consumption, mouse

## Abstract

Successful pregnancy for optimal fetal growth requires adequate early angiogenesis and remodeling of decidual spiral arterioles during placentation. Prior to the initiation of invasion and endothelial replacement by trophoblasts, interactions between decidual stromal cells and maternal leukocytes, such as uterine natural killer cells and macrophages, play crucial roles in the processes of early maternal vascularization, such as proliferation, apoptosis, migration, differentiation, and matrix and vessel remodeling. These placental angiogenic events are highly dependent on the coordination of several mechanisms at the early maternal–fetal interface, and one of them is the expression and activity of matrix metalloproteinases (MMPs) and endothelial nitric oxide synthases (NOSs). Inadequate balances of MMPs and nitric oxide (NO) are involved in several placentopathies and pregnancy complications. Since alcohol consumption during gestation can affect fetal growth associated with abnormal placental development, recently, we showed, in a mouse model, that perigestational alcohol consumption up to organogenesis induces fetal malformations related to deficient growth and vascular morphogenesis of the placenta at term. In this review, we summarize the current knowledge of the early processes of maternal vascularization that lead to the formation of the definitive placenta and the roles of angiogenic MMP and NOS/NO mechanisms during normal and altered early gestation in mice. Then, we propose hypothetical defective decidual cellular and MMP and NOS/NO mechanisms involved in abnormal decidual vascularization induced by perigestational alcohol consumption in an experimental mouse model. This review highlights the important roles of decidual cells and their MMP and NOS balances in the physiological and pathophysiological early maternal angiogenesis–vascularization during placentation in mice.

## Introduction

Maternal uterine microvascular adaptations during pregnancy are critical for optimal intrauterine growth, fetal survival (Creeth and John, 2020), and postnatal life. Placenta, a specialized transient organ during pregnancy, is responsible for keeping up with fetal nutritional demands and oxygen, molecule, and hormone feto–maternal exchanges (Guttmacher et al., 2014; Martín-Estal et al., 2019). During the formation of the hemochorial placenta, *de novo* maternal vascularization is dependent on not only normal development and function of trophoblast cells but also adequate angiogenesis in decidual tissue. During decidualization, early maternal vascularization involves physiological modification, branching, and growth of the uterine artery to transform into spiral arterioles (SAs). The maternal angiogenesis and remodeling of the decidual vascular network, consisting of low-resistance vessels to transport maternal blood with high volume flow at low velocity and pressure, is coordinated by decidual stromal cells, including uterine natural killer (uNK) cells and macrophages (Rätsep et al., 2015). The initiation of SA and matrix remodeling coincides with leukocyte infiltration and residence in the decidua. Decidual NK cells and macrophages, of great significance in early angiogenesis and placental development to pregnancy maintenance (Qin et al., 2023), express relevant factors, including metalloproteinases (MMPs) (Choudhury et al., 2019) and nitric oxide synthases (NOSs), which are some of the most important regulatory complex pathways of angiogenic-remodeling mechanisms of decidual tissue during early gestation.

Imbalanced expression, activity, and release of angiogenic and vasoactive mediators can lead to inadequate spiral artery angiogenesis, such as low decidual vascular remodeling and vasoconstriction of the spiral arteries during the early placentation period, which subsequently reduce perfusion and increase pressure in the placental feto–maternal interface (Gyselaers, 2023). An abnormal maternal angiogenesis and vascular function consequently can induce placental vascular malperfusion, insufficiency, hypertension, vasculopathy, and anatomical placental disorders, leading to gestational complications, recurrent spontaneous abortion, preeclampsia, and preterm labor (Reynolds et al., 2006; Khong et al., 2016; Gyselaers, 2023). Currently, placental abnormalities are associated with perinatal fetal malnutrition, morbidity, mortality, and fetal growth restriction (Sharma et al., 2016; Lacko et al., 2017; Perez-Garcia et al., 2018; Woods et al., 2018) and predispose to programming diseases in adults, such as coronary heart disease, type 2 diabetes mellitus, hyperinsulinemia, and the risk of developing adult-onset degenerative diseases (McMillen and Robinson, 2005; Hanson and Gluckman, 2008).

Different reports suggest that fetal and postnatal defects induced by alcohol use and abuse in gestation, including “fetal alcohol spectrum disorders” (FASDs) (Gupta et al., 2016; Hoyme et al., 2016), adulthood obesity, metabolic syndromes, and cardiovascular disease, among many others, may be due to placental abnormalities (Bada et al., 2005; Burd et al., 2007; Aliyu et al., 2008; 2011; Gundogan et al., 2008; 2010; 2015; Meyer-Leu et al., 2011; Patra et al., 2011; Salihu et al., 2011; Ramadoss and Magness, 2012; Avalos et al., 2014; Lui et al., 2014; Zhu et al., 2015; Carter et al., 2016; Linask and Han, 2016; Davis-Anderson et al., 2017; Tai et al., 2017; Ohira et al., 2019; Orzabal et al., 2019; Kwan et al., 2020; Odendaal et al., 2020). Some studies, both in human and animal models, have focused on the effects of maternal alcohol ingestion on the vascularization of the definitive placenta. Gestational alcohol exposure can alter maternal systemic and reproductive circulatory adaptations during pregnancy, affecting uteroplacental vascular hemodynamics with induced vascular resistance, among other gestational variables. Gestational alcohol intake produces the “alcohol-related placental-associated syndrome” (Salihu et al., 2011) that includes miscarriage, hypertension, preeclampsia, preterm birth, placenta previa, placenta accreta, and placental hemorrhage (Gundogan et al., 2010; Avalos et al., 2014; Carter et al., 2016; Tai et al., 2017; Ohira et al., 2019; Orzabal et al., 2019; Odendaal et al., 2020). Moreover, a high risk of placental abruption was observed after the consumption of 7–21 drinks per week (a mean of two drinks per day and BAC of 5–100 mg/dL) (Burd et al., 2007). Chronic gestational alcohol in rats (37% of caloric content) impairs the physiological remodeling of the maternal placental vasculature (Ramadoss and Magness, 2012). Related to this, some altered processes proposed to explain uteroplacental vascular dysfunction following gestational alcohol consumption were the disruption of trophoblastic function, such as inadequate or poor differentiation, migration/invasion (Han et al., 2012), and defective remodeling/endothelial replacement of maternal vessels by these cells (Radek et al., 2005; Rosenberg et al., 2010; Ramadoss and Magness, 2012b; 2012c; Subramanian et al., 2014; Orzabal et al., 2019). Gundogan et al. (2008, 2010) reported, in an animal model, that one major placental abnormality due to chronic gestational ethanol exposure is the failure of maternal decidual spiral artery remodeling by invasive trophoblasts, thus leading to altered placental blood flow and nutrient exchange. A moderate or high dose of ethanol intake during gestation also reduces labyrinthine development (Gundogan et al., 2008; 2010; 2015). During the third trimester of gestation, alcohol affects the uteroplacental vascular function (Rosenberg et al., 2010; Subramanian et al., 2014; Orzabal et al., 2019) by impairment of uterine spiral artery remodeling, angiogenesis, and vasodilation (Radek et al., 2005) via altered endothelial angiogenic gene expression (Ramadoss and Magness, 2012b; 2012c). Table 1 summarizes the present background of altered placental vascularization and some mechanisms involved, following gestational alcohol exposure in rat, mouse, and human models. However, although early angiogenesis and vessel remodeling are major contributing processes defining normal placental circulation, at present, there are few studies examining the earlier etiology and molecular mechanisms involved in maternal alcohol-induced vasculopathy of the placenta in the mouse model.

**TABLE 1:** Summary of the effects on placental vascularization produced by maternal gestational alcohol exposure.

Alcohol exposure pattern	Ethanol exposure values	Time of exposure	Placental vascularization effects	References
Rat model				
Chronic alcohol gestational exposure	8%–18%–24%–37% EDC	Gestational days 6–16	Placental oxidative stress and mitochondrial dysfunction	Gundogan et al. (2008, 2010), Gundogan et al. (2015)
			Incomplete maternal artery remodeling	Ramadoss and Magness (2012)
			Diminished placental blood flow	
			Placental vasoconstriction hypoxia	
			Altered labyrinthine layer	
			Disrupted spiral artery vascular muscular layer	
			Impaired physiological conversion of the maternal uterine vasculature	
Chronic alcohol perigestational exposure	10%–30% v/v ethanol	1 month before and during gestation	Decreased (52%) placental blood flow	Ramadoss and Magness (2012)
Chronic alcohol gestational exposure	4.5 g/kg dose (BAC: 216 mg/dL)	Gestational days 5–19	Impaired production and NO pathway in uterine artery	Orzabal et al. (2019)
	(daily orogastric gavage)		Impaired uterine vascular remodeling	
			Reduced uterine artery eNOS expression and activity	
			Decreased uterine vasodilation	
			Upregulated MMP-28, RT1-M6-2, MMP-2, and MMP-9	
			Diminished uterine artery function and altered proteome	
Chronic alcohol gestational exposure	4.5 g ethanol/kg/day	Gestational days 7–17	Impaired maternal uterine artery reactivity	Subramanian et al. (2014)
(chronic binge-like alcohol)	(BAC: 216 mg/dL)		Decreased uterine vasodilation	
			Uterine vascular dysfunction	
			Impairs agonist-induced uterine artery vasodilation	
			Impaired acetylcholine-mediated uterine artery vasodilation	
**Mouse model**				
Acute alcohol gestational exposure	Two i.p. injections 3 g/kg ethanol	Gestational days 8–8.75	Reduced late placental labyrinth	Haghighi Poodeh et al. (2012)
			Altered cell junctions of placental barrier	
			Increased permeability	
			VEGF upregulation in 9.5, 11.5, and 14.5 dpc-placenta	
			Premature permeability of placenta–yolk and reduced VEGF	
			Altered permeability and malfunction of yolk sac tissues	
Chronic alcohol gestational exposure	(BAC 110 mg/dL)	Gestational days 6–18	Placental resistance	Ramadoss and Magness (2012)
			Abnormal vascular perfusion	
			Reduced NO modulation of the mesenteric artery	
			Reduced maximal relaxation response to stimulation	
**Human model**				
Chronic alcohol gestational exposure	20 g ethanol/day	During early gestation	Diminished placental vascular density	Holbrook et al. (2019)
	(∼10 standard drinks/week)		Decreased KDR expression in placenta at term	
Semi-chronic alcohol gestational exposure	<1.5 or +1.5 drinks/week	Until the second/third trimester	Placental abnormality and altered NO	Ohira et al. (2019)
	(Standard drink = 14 g ethanol)		Placenta accreta	
	(Drinking frequency: <3 or 3+ days/week)			
Chronic alcohol gestational exposure			Altered maternal uterine artery transcriptome	Ramadoss and Magness (2012)
			Altered transcription of proteases/matrix proteins	
			Altered uterine vascular remodeling	
			Altered uterine angiogenic mRNA transcriptome	
Chronic alcohol gestational exposure	Moderate–high concentrations	Along gestation	Impaired blood flow/artery vasodilatation	Burd and Hofer (2008)
			Abnormal nutrient transport	
			Umbilical cord vasoconstriction	
Chronic alcohol gestational exposure	Average of 200–250 mL ethanol/trimester	Along gestation	Altered contractility of the umbilical cord artery	Iveli et al. (2007)
			Altered vasculature, uteroplacental malperfusion, and resistance	
			Inhibition of NO release and prostacyclin	
Chronic–semi-chronic alcohol gestational	Heavy, moderate, and/or light	2nd, 3rd, 1st + 3rd or	Uteroplacental malperfusion and hypoplasia	Tai y col. 2017
	drinking	2nd + 3rd trimesters	Premature delivery, IUGR	
Acute alcohol gestational exposure	Eight drinks (one binge-heavy)	1.5 days/week during gestation	Decreased placental growth	Carter et al. (2016)
Chronic alcohol gestational exposure	Two drinks (wine)/day	Along gestation	Placental abruption, IUGR	Burd et al. (2007)
	(18–30 g ethanol/day)		Abnormal fetus	
	(BAC 5–100 mg/dL)			

The ethanol values are given in g, % intake, BAC, % EDC, drinks. i. p.: intraperitoneal; EDC: ethanol-derived calorie; IUGR: intrauterine growth restriction; BAC: blood alcohol concentration.

However, maternal alcohol consumption is often associated with chronic alcohol ingestion by women prior to and up to early gestation (around 4–6 weeks after human conception). It is common to find a high proportion of female consumers that continue to drink moderate quantities of alcohol (200 mL/day of wine containing ethanol 11%) up to early gestation, while being unaware that they are pregnant. In this regard, some data indicate that approximately 47% of women drink around conception, and 15%–39% cases reported consumption of high doses of alcohol (more than five standard drinks on one occasion) (Colvin et al., 2007). In this context, periconceptional alcohol exposure in the highly susceptible peri-implantatory and early organogenic periods may be affected and can lead to placentopathy at term (Gualdoni et al., 2022b). In view of these periods of consumption, we established a mouse model of perigestational alcohol consumption starting before pregnancy and continuing up to early gestation (mouse gestational days (GD) 4, 5, 7, 8, and 10) to study the embryo development (Cebral et al., 2007; 2011; Coll et al., 2011; 2017; Perez-Tito et al., 2014; Gualdoni et al., 2022a). We have shown that moderate oral ethanol intake for 2 weeks before pregnancy and up to peri-implantational stages affect embryo differentiation and growth and leads to retardation during implantation (Pérez-Tito et al., 2014). In addition, PAC up to early mouse organogenesis (day 10 of gestation) induces delayed embryo development, reduces viability, produces dysmorphogenesis of the neural tube, deregulates the embryonic cadherin expression (Coll et al., 2011), alters the arachidonic acid metabolic pathways (Cebral et al., 2007), and generates embryo oxidative stress (Coll et al., 2017), among other effects. Nevertheless, to date, little is known about the effects of PAC on early maternal vascularization during placentation. Recently, we suggested that one cause of mouse placental abnormalities found at term after PAC (Gualdoni et al., 2022b) may be associated with alterations in decidual and trophoblast tissue development during exposure up to organogenesis (Coll et al., 2018; Ventureira et al., 2019; Gualdoni et al., 2021). Apart from imbalances in the endothelial vascular endothelial growth factor (VEGF) system, defects in MMP and NOS expression/activity and levels of NO may also partially explain the early abnormal placentation (Gualdoni et al., 2021). However, at present, there is little evidence, in animal models, of the deleterious effects of maternal alcohol exposure on early decidual angiogenesis–vascularization and the involvement of MMP and NOS/NO factors as mechanisms responsible for triggering late-stage placental abnormalities.

Considering the background and importance of early maternal vascularization of the placenta for optimal fetal growth and maintenance and successful pregnancy at term, and subsequent life course, in this review, we first overview the current knowledge of early decidual vascular angiogenesis and remodeling involved in normal and abnormal mouse early placentation, focusing on the roles of MMP and NOS/NO systems. Then, we propose hypothetical altered decidual cellular and MMP and NOS/NO mechanisms involved in early maternal abnormal vascularization of the placenta in a PAC experimental mouse model. Overall, this review highlights the importance of adequate early gestational balances of decidual cells and MMPs and NO in the physiology and pathophysiology of maternal vascular development during placentation.

## Metalloproteinases and nitric oxide as angiogenic placental mechanisms

During the early period of mouse pregnancy, decidual capillaries and arterioles develop by angiogenesis, the process of new blood vessel formation and growth from pre-existing vessels. Angiogenesis involves 1) the expression of angiogenic factors, 2) vascular growth stimulation by hypoxia, 3) secretion of essential proteases for tissue remodeling, 4) migration of endothelial cells, and 5) appropriate proliferation of endothelial cells to secure the outgrowth of a new vessel. Disruption of the balance between angiogenic factors and their inhibitors may result in early miscarriage or, alternatively, defective placentation, thereby increasing the risk of pregnancy-related disorders.

The processes of neovascularization require the response to a complex paracrine network of factors (Adams and Alitalo, 2007; Bryan and D’Amore, 2007; Hellstrom M et al., 2007), of which one of the most important is the expression and activation of the VEGF system that involves in a proper gradient necessary to stimulate sprouting and branching of maternal vessels (Bautch, 2012; Gualdoni et al., 2022b). Imbalances of the VEGF lead to aberrant placental vascular morphogenesis (Roberts and Escudero, 2012; Li et al., 2014).

In arteriolar vascular smooth muscle and periendothelial cells of capillaries, the VEGF can act through three receptors: VEGF-R1 (FLT-1), VEGF-R2 (KDR/Flk-1), and VEGF-R3 (FLT-3). The main physiological VEGF effects are via the activation of KDR (Chung and Ferrara, 2011), which induces downstream activation of signaling cascades that stimulate the production of at least 11 angiogenic factors (Lima et al., 2014; Apte et al., 2019), including the placental angiogenic MMP and eNOS regulators (Kimura and Esumi, 2003).

MMPs are multigenic proteolytic zinc-dependent enzymes composed of six classes: collagenases, gelatinases, stromelysins, matrilysins, and membrane-type MMPs, among others (Amălinei et al., 2007; Cui et al., 2017; Henriet and Emonard, 2019). The expressions of MMP-1, MMP-2, MMP-3, MMP-7, MMP-8, MMP-9, MMP-10, MMP-13, and MMP-19 are increased by the activation of the VEGF system (Chen and Khalil, 2017). VEGF-KDR activation upregulates MMP-2 and MMP-9 expressions in human umbilical vein endothelial cells (Heo et al., 2010), and FLT-1 can modulate KDR to regulate the expression of MMP-9 to prevent excessive angiogenesis (Wang and Keiser, 1998). MMPs, secreted as zymogens, are strictly controlled at transcription, secretion, and activation/inhibition levels by exogenous and endogenous factors, such as cytokines, growth factors, hormones, their inhibitors (tissue inhibitors of metalloproteinases (TIMP), oxidative stress, phosphorylation, hypoxia-re-oxygenation, transcription factors, and others (Nuttall et al., 2004; Amălinei et al., 2007; Jacob-Ferreira and Schulz, 2013; Cui et al., 2017). Activation of MMP-2 can occur by non-proteolytic post-translational modifications of the full-length zymogen, by S-glutathiolation, S-nitrosylation, and phosphorylation (Jacob-Ferreira and Schulz, 2013). Post-translational activation of latent forms of MMPs is generally processed in the extracellular compartment (Henriet and Emonard, 2019). Three tissue inhibitors for MMP (TIMP-1, TIMP-2, and TIMP-3) regulate MMP activity. TIMP-1 forms complexes specifically with MMP-9 and TIMP-2 is involved in the regulation of MMP-2 activity. Interestingly, TIMP-3 supports the activation of MMP-2 via membrane-type MMP, as well as inhibition (Alexander et al., 1996). On the other hand, TGFβ1, expressed in the mouse endometrium during implantation and decidualization, has a potential role in the regulation of MMP-9 gene expression (Bany et al., 2000).

MMPs have multiple roles, such as tissue and extracellular matrix (ECM) remodeling, proliferation, apoptosis, migration, differentiation, invasion (Hamutoğlu et al., 2020), cell–matrix and cell–cell interactions, and activation or inactivation of autocrine or paracrine signaling molecules (Amălinei et al., 2007). In addition, proteolysis of the ECM could release matrix-bound growth factors and their receptors. MMPs can directly activate growth factors, and MMP-1, MMP-2, MMP-3, MMP-7, and MMP-9 activate TNFα (Hulboy et al., 1997).

Placental hemodynamics and adaptations require extensive structural and functional modifications in the maternal blood vessels of the placenta. In normal pregnancy, remodeling of maternal vessels needs secretion of MMPs by trophoblast and decidual stromal cells to mediate proteolysis and degradation, vascular and matrix remodeling, and angiogenesis (Staun-Ram et al., 2004; Gualdoni et al., 2022; Rusidzé et al., 2023). Through their proteolytic activity, MMPs may mediate detachment of pericytes from the vessels, release ECM-bound angiogenic growth factors, expose cryptic proangiogenic integrin-binding sites in the ECM, generate promigratory ECM component fragments, and cleave endothelial cell–cell adhesions (Rundhaug, 2005). The most important gelatinases with a preponderant role in maternal tissue remodeling, degradation of the basement membrane and ECM (Cui et al., 2017), vasodilatation, and uterine expansion during normal pregnancy in humans and mice are MMP-2 and MMP-9 (Staun-Ram et al., 2004; Fontana et al., 2012; Chen and Khalil, 2017). Specifically, MMP-9, which degrades type IV, V, and IX collagens, gelatin, and elastin, is involved in numerous processes, including implantation, placentation, and embryogenesis (Silvia and Serakides, 2016; Espino et al., 2017; Quintero-Fabián et al., 2019; Timokhina et al., 2020). MMP-9 has been shown to be involved in endometrial remodeling in mouse invasion of trophoblasts (Zhang et al., 2020), development of mouse decidua, vascularization of the implantation site during early organogenesis (Fontana et al., 2012; Gualdoni et al., 2021), and remodeling of the feto–maternal interface during advanced stages of mouse placentation (Rusidzé et al., 2023). However, little has been reported about the localization, expression, and activation of MMP-2 and -9 in the early mouse implantation site prior to the formation of the placenta. In mice, by GD6 and GD8, MMP-9 immunoreactivity was found mainly in the antimesometrial non-decidualized endometrium and in trophoblast giant cells (Alexander et al., 1996). However, deciduomas, in the absence of trophoblast cells, were shown to contain the same level of MMP-9 activity as decidua (Bany et al., 2000), suggesting that this protease can be present in decidua derived from other than decidual cells. Moreover, maternal expression of MMP-9 was demonstrated to be increased in the uterus during decidualization and in cultured mouse endometrial stromal cells from uteri sensitized for decidualization. At mid-gestation, MMP-9 transcripts and protein were found in trophoblast cells (Teesalu et al., 1999; Gualdoni et al., 2021), stromal cells, and the ECM of mesometrial decidua (Fontana et al., 2012), suggesting its role in the angiogenesis of maternal tissue during mouse organogenesis. On the other hand, at mouse gestational day 6, MMP-2 is expressed and activated in the endometrium (Alexander et al., 1996; Bany et al., 2000); by day 8, it was detected in stromal cells at the mesometrial pole of the mouse implantation site and also in the area of the primary trophoblast giant cells, while from mouse mid-pregnancy (GD10), MMP-2 was expressed in trophoblast and decidual tissue (Teesalu et al., 1999; Heo et al., 2010; Gualdoni et al., 2021).

Altered MMP-2 and MMP-9 expression/activity could lead to the release of inflammatory cytokines, hypoxia-inducible factor, and reactive oxygen species, which may target the ECM and endothelial and vascular smooth muscle cells, causing inadequate remodeling of spiral arteries, generalized vascular dysfunction, increased vasoconstriction, and placental ischemia, in turn contributing to the pathogenesis of abnormal gestational outcome, such as premature birth, hypertension, and complications of pregnancy (Jacob-Ferreira and Schulz, 2013; Chen et al., 2020). Particularly, MMP-9 deficiency in homozygous MMP-9 knockout mice was associated with reduced and abnormal development of ectoplacental cones at GD 7.5, with impaired trophoblast differentiation and reduced invasion (Plaks et al., 2013). Also, in MMP-9 null mice, MMP-9 deficiency was associated with a decrease in the number of pregnancies and a reduction in litter size (Dubois et al., 1999; Dubois et al., 2000).

Nitric oxide, the other relevant angiogenic–vasoactive factor, is produced by the oxidation of L-arginine catalyzed by three NOS enzyme isoforms: neuronal (nNOS or NOS1), inducible (iNOS or NOS2), and endothelial (eNOS or NOS3) (Hefler et al., 2001; Förstermann and Sessa, 2012; Qian and Fulton, 2013). The nNOS and eNOS isoforms are frequently expressed constitutively, and their activities are regulated by calcium availability, whereas iNOS is independent of the intracellular calcium concentration and generates a high flow of NO (Förstermann and Sessa, 2012; Eelen et al., 2017). nNOS and iNOS are predominantly cytosolic, whereas eNOS can be either cytosolic or localized in membrane caveolae of endothelial cells (Eelen et al., 2017). In normal pregnancy, regulated by the VEGF by increasing the endothelial calcium signaling, eNOS is the most relevant enzyme in NO production (Moncada and Higgs, 2006). Additionally, endothelial shear stress, produced by flowing blood, can stimulate endothelial NO release and increase endothelial intracellular free Ca^2+^ concentration (Vanhoutte et al., 2016). Under basal conditions, iNOS is normally not expressed. However, different stimuli, including immunologic and inflammatory cues, can induce iNOS expression in various cell types under changes in the cellular environment (Eelen et al., 2017).

Endothelial NO, acting on vascular smooth muscle cells (VSMCs), is a major local regulator of vasodilatation and blood flow (Chen and Khalil, 2017). In the endothelium, the primary function of NO is to relax vascular smooth muscle tissue and regulate arterial blood pressure (Waker et al., 2023). However, NO also plays roles in angiogenesis, such as endothelial cell (EC) migration, control of microvascular volume, vascular permeability, platelet aggregation, and thrombosis (Krause et al., 2011; Aban et al., 2013). During gestational vascularization, new EC function depends on NO release from pre-existing ones, while the vascular smooth muscle responds to released NO levels paracrinally (Esper et al., 2006). Under controlling by oxygen tension, hormones, and oxidative stress, among others, NO supports placental vascularization (Krause et al., 2011) to maintain vascular vasodilatation and low vascular resistance (Boeldt et al., 2011) and to attenuate the effects of vasoconstrictors at the feto–placental interface (Possomato-Vieira and Khalil, 2016).

Relevant balance and gradual increase of the levels of NO are needed for normal gestation, as demonstrated in eNOS knockout mice, where the spiral artery remodeling was dysregulated, the labyrinth was reduced, and uteroplacental hypoxia was induced (Kulandavelu et al., 2012; Kusinski et al., 2012; Walker et al., 2023 **).**

### Brief overview of early maternal vascularization during mouse placentation

The mouse placenta contains three main layers: the maternal component of the decidua, the fetal-derived junctional zone (JZ), and the labyrinth (Lab). The decidua, playing a major role in placental development, is derived from decidualization of uterine endometrial stroma. The JZ is composed of glycogen trophoblast cells (GlyTs), spongiotrophoblast cells (SpTs), and parietal trophoblast giant cells (P-TGCs) (Simmons et al., 2007). The placental labyrinth consists of an anastomosing network of interdigitated maternal blood sinusoids and fetal capillaries (Rusidzé et al., 2023). To establish the definitive placenta, two vascular systems are crucial to develop sequentially during mouse placentation: the maternal vasculature, consisting mainly of decidual spiral arteries, and the labyrinth (Woods et al., 2018). For achieving normal and adequate vascularization and normal labyrinthine formation in the definitive placenta, important changes must operate in the development of the maternal circulatory system during early pregnancy (Torry et al., 2007).

To meet the needs of the growing embryo and maximize placental perfusion and maintenance of efficient maternal and fetal exchange, maternal vessels are transformed into large-caliber ones in a process called *spiral artery remodeling,* which results in decreased maternal vascular resistance (Prefumo et al., 2004; Mu and Adamson, 2006) and increased placental vascular diameter and elasticity (Mu and Adamson, 2006; Burton et al., 2009). In early gestation, for normal maternal vascularization, complex angiogenic processes of remodeling take place in decidual tissue by which the uterine spiral arteries extend, branch, lose the muscular layer, and remodel to become dilated (Mandala and Osol, 2011; Brosens et al., 2019). In mice, similar to humans (Smith et al., 2009), angiogenesis-remodeling of maternal vasculature may occur in two phases: the trophoblast-independent phase during early gestation, where maternal angiogenesis depends on cells of the decidua; and the trophoblast-dependent phase, occurring during the development of the definitive placenta, from around mouse mid-gestation (GD 10.5–11), when trophoblasts, in coordination with decidual cells, play a major role in completing the remodeling of maternal decidual vessels at the JZ (Cross et al., 2002; Rai and Cross, 2014; Rusidzé et al., 2023).

Although the process of SA remodeling by trophoblast cells in mouse mid-gestation is described very well (Rusidzé et al., 2023), to date, little emphasis has been given in the literature to the maternal decidual tissue and spiral arteriole remodeling and angiogenesis in the decidua earlier in the mouse implantation site. To provide insight into early maternal vascularization, we next summarize the main events of decidual angiogenesis, with emphasis on the first phase of SA remodeling.

### Trophoblast-independent phase of maternal angiogenesis–vascularization

**FIGURE 1:**
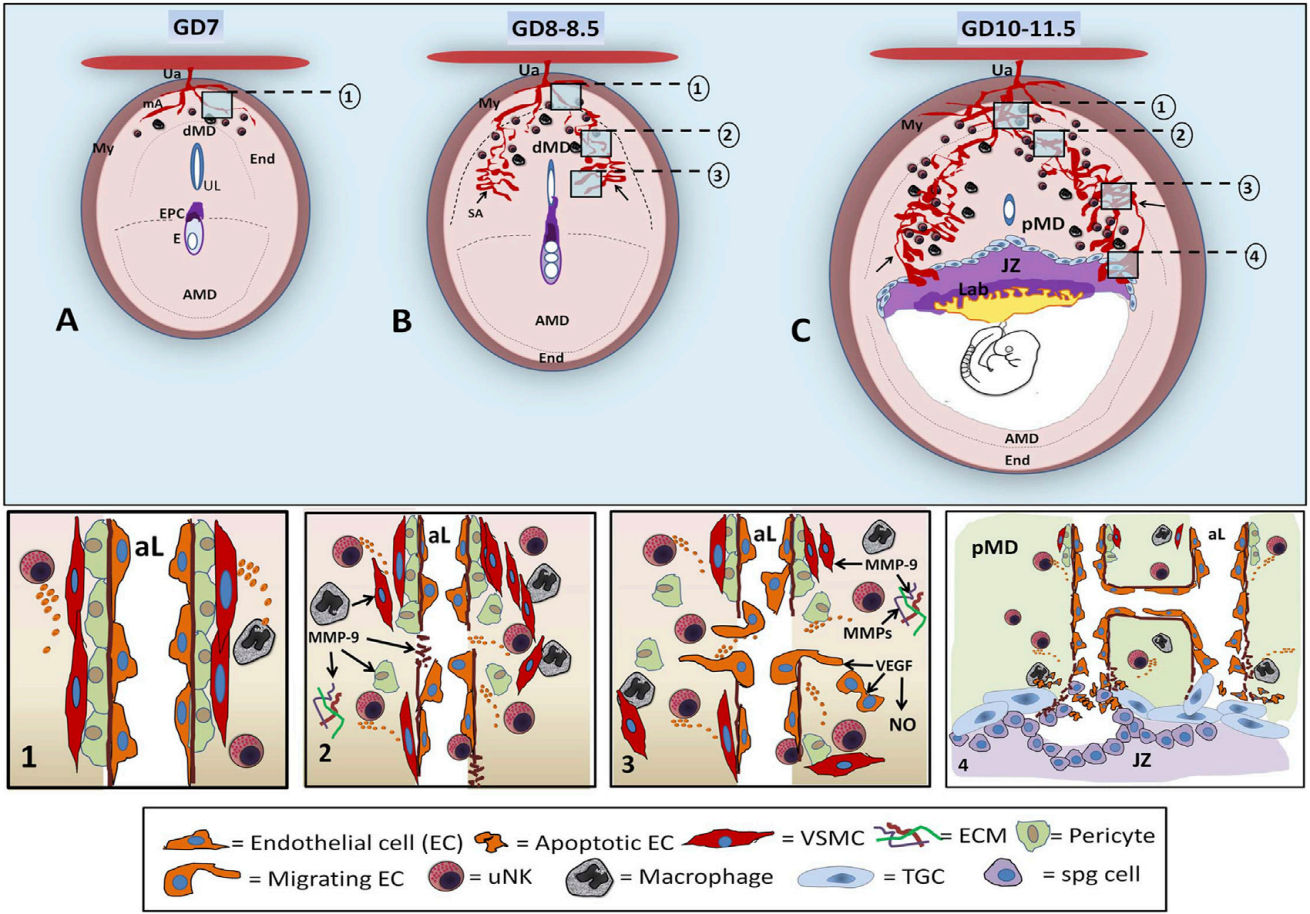
Diagrams of early decidual vascularization in the mouse implantation site. In the mouse implantation site (IS) of GD 7, the mesometrial uterine artery (Ua) crosses the myometrium (My), reaches the endometrium (End), and branches in maternal arterioles (ma) in the distal mesometrial decidua (dMD), where few uNKs and macrophages can be found **(A)**. At GD 8–8.5, angiogenesis-remodeling leads to increase in the lumen of decidual SAs (arrow) at the lateral sides of mesometrial decidua with respect to the center of the uterine lumen (UL) and ectoplacental cone (EPC). uNKs and macrophages increase in number in MD **(B)**. By GD 10–11.5, ramified, dilated, and partially remodeled SAs, seen at lateral regions of the proximal mesometrial decidua (pMD), converge at the JZ, forming sinusoidal maternal lacunae that will have contact with the Lab. At this stage, peak uNKs are observed in the pMD. The decidual SA-angiogenesis and remodeling involve several steps (numbered inserts in the upper diagrams of ISs are shown in the bottom panels). The unremodeled SAs are characterized by an intact typical wall with pericytes and VSMCs and ECs [A, step **(1)**]. In dMD, uNKs and macrophages begin to secrete angiogenic factors around the SAs, inducing maternal vessel destabilization and leading to the separation of VSMCs and pericytes, basement membrane rupture, permeability, ECM remodeling, and disruption of CE junctions [B, step **(2)**]. At the pMD, sprouting angiogenic events consist of EC migration and proliferation, VSMC remodeling, increased permeability, and maternal blood extravasation [B, step **(3)**]. At GD10–11.5 **(C)**, tubulogenesis in the pMD involves EC recruitment, EC adhesion and fusion, basement membrane formation, ECM remodeling, and SA elongation and ramification, yielding to vessels with large luminal extensions at the lateral side of IS [C, step **(4)**]. The SAs arrive at the JZ partially remodeled (without or with few VSMCs), where invasive JZ trophoblasts, derived from spongiotrophoblast (spg), cause apoptosis of maternal EC to eventually replace the maternal endothelium and to form a pseudo-endothelium lined by trophoblastic cells. Trophoblasts, uNKs, and macrophages establish a crosstalk that plays a role via the expression of VEGF, MMPs, NOS, and other factors, in the definitive SA remodeling in the placenta (AMD: antimesometrial decidua).

In mice, the maternal vascular tree originates from the uterine artery, which branches into radial arteries and then into spiral arterioles (SAs). Early, in GD 7–7.5, on the mesometrial side of the mouse implantation site, uterine radial arteries are detected in the myometrium, from where they begin to branch into the arcuate arteries and into radial arteries in the distal region of decidua of the implantation site (Figure 1A). At GD 8–8.5, several spiral arterioles (SAs) exhibit a narrow lumen, indicating their low development at this stage (Figure 1B). At mid-gestation (GD 10–11), branched and dilated SAs form a very dense capillary plexus and maternal blood lacunae at the mesometrial decidua (Cross et al., 2002; Ventureira et al., 2019), which converge at the junctional zone, to be then remodeled completely by trophoblastic cells and form maternal sinusoidal lacunae surrounded by trophoblastic tissues in the new placenta (Croy et al., 2013) (Figure 1C).

Prior to the establishment of the functional mouse choriovitelline placenta (Mu and Adamson, 2006), and previous to maternal vascular remodeling by trophoblast cells at the feto–maternal interface, angiogenesis of maternal vessels is mediated by stromal and immune cells of decidual tissue through the promotion of many decidual growth and vasoactive factors, cytokines, and others (Croy et al., 2003; 2009; 2012; Ramathal et al., 2010). This early trophoblast-independent phase of remodeling is characterized by the onset of decidualization and leukocyte infiltration of the spiral artery wall, MMP secretion, disruption of the VMSC layer, and vascular cell detachment and loss.

To achieve normal maternal vascularization at the mesometrial side of the mouse implantation site, decidualization of the uterine endometrium is the first important event that should occur. Mouse decidualization begins immediately following implantation, when the antimesometrial endometrial fibroblast cells start to proliferate and differentiate into the large epithelioid, binucleated decidual cells (GD 6–6.5), to form the primary decidual zone. This densely packed avascular decidua grows dramatically and extends toward the mesometrial compartment of the implantation site (GD 7.5) (Dey et al., 2004; Wang et al., 2004), while the extracellular matrix of the endometrial stroma is remodeled, including modifications in collagen fibril distribution, structure, and thickness (Carbone et al., 2006). Decidualization is an important event to immediately prepare the maternal tissue for angiogenic processes to occur properly.

At an early stage, at GD7 (Figure 1A), the unremodeled intact maternal vessels (Figure 1A Step 1) respond to the hypoxia state and several EC- and decidual stromal cell-secreting angiogenic gradient of factors (Distler et al., 2003; Valdes et al., 2008; Blois et al., 2011; Zhang et al., 2011; Lima et al., 2014; Grochot-Przeczek et al., 2015; Sojka et al., 2019). From this stage of gestation and further, the main steps of decidual angiogenesis–vascularization involve 1) vessel destabilization, consisting of detachment and removal of the arterial wall, de-differentiation of VMSCs, and ECM remodeling by MMPs, which promotes the release of growth factors (bFGF, VEGF, and IGF-1) sequestered in the ECM. Also, enzymatic degradation of the endothelial basement membrane and pericyte cell and EC detachment can be attributable to MMP actions. Vascular permeability begins to increase in response to hypoxia-stimulation of the VEGF, directing the associated expression of NOS and subsequent increment of NO and then allowing extravasation of plasma proteins that lay down a provisional scaffold for migrating endothelial cells (Figure 1B; Figure 1 Step 2). 2) Vascular sprouting involves EC detachment, ECM remodeling by MMPs, CE migration induced by loosening interendothelial cell contacts and mechanical stress, CE proliferation in the ECM (Lamalice et al., 2007) induced by VEGF-A, and increase of permeability and extravasation of maternal blood, directed by NO (Figure 1B; Figure 1 Step 3). 3) Tubulogenesis consists of EC and pericyte recruitment and adhesion into new vessels; basement membrane formation; ECM remodeling; and EC differentiation and adhesion (Figure 1 Step 4). Tubulogenesis implies SA branching, elongation, anastomosis, vessel looping, and vasodilatation, processes that yield large luminal extensions of maternal decidual vessels at the lateral side of the mouse implantation site already to GD 10 (Figure 1C; Figure 1 Step 4).

**FIGURE 2:**
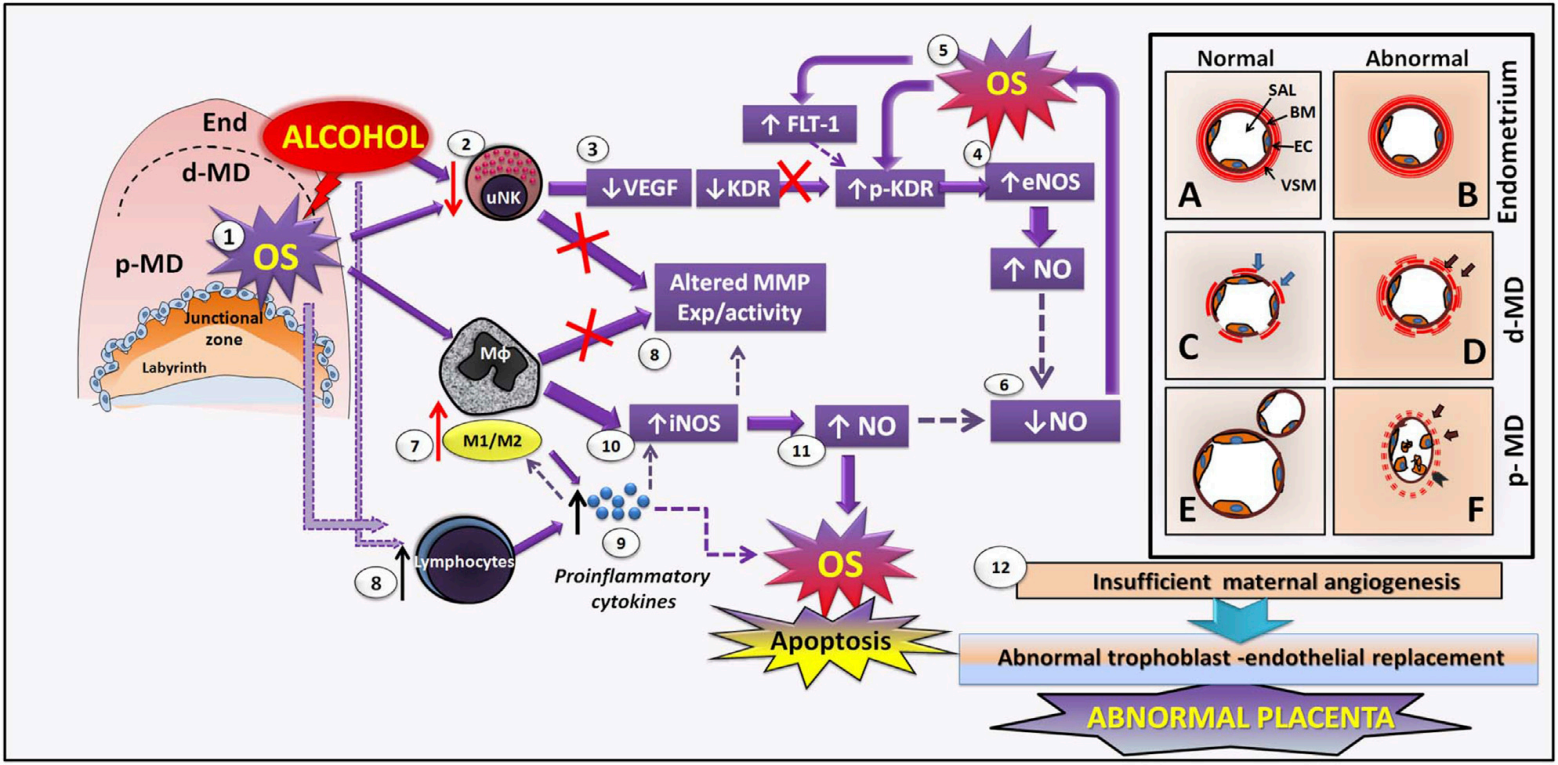
Hypothetical mechanisms of early maternal vascular disruption and altered MMP–NO pathways induced by perigestational alcohol consumption in an experimental mouse model. After PAC, alcohol directly impacts decidual tissue and maternal vasculature, producing OS (1), by which uNKs are diminished (2). In consequence, uNK–VEGF production is also reduced (3). Alcohol and OS induce low KDR expression and KDR activation (phosphorylated pKDR), which conduces to eNOS activation in endothelial cells (CE) and decidual cells (4), producing high levels of NO that enhances OS (5). Thus, bioavailability of NO is reduced in maternal vasculature (6) after PAC, perhaps leading to reduced proliferation and angiogenesis and decidual artery vasoconstriction. PAC-induced OS can be involved in macrophage phenotype change, increasing the inflammatory ratio M1/M2 (7) disrupting the expression (exp) and activity of MMPs (8). In parallel, alcohol and OS may induce increase of lymphocytes (8), which release high levels of proinflammatory cytokines (9) that enhance the balance of M1/M2macrophages in the decidua (7). Proinflamatory macrophages may activate iNOS (10) yielding to increase NO (11). High NO enhances OS and apoptosis in maternal vasculature. Altered macrophagic MMPs and NO contribute to abnormal ECM and CE remodeling, leading to defective decidual vascularization. While there are not differences between normal and abnormal decidual vasculature in non-decidualized endometrium **(A, B)**, in the distal mesometrial decidua (dMD), the abnormal vasculature **(D)**, associated to PAC, consist on maternal vessels with uncompleted remodelled vascular smooth muscle (VSM) cells in the vascular wall (black arrows) and less numbers of endothelial cells (EC), compared to the normal maternal vessel **(C)** in control condition. Then, in proximal mesometrial decidua (pMD), abnormal maternal vessels exposed to PAC have unremodelled VSM (arrows), disrupted endothelium and increased free EC apoptosis into the lumen, and reduced maternal vascular expansion **(F)** compared to normal pMD-vasculature **(E)** which spiral arteries are completely remodelled, expanded and ramified. Overall, disrupted SA angiogenesis in early decidua may result in insufficient maternal artery transformation and subsequent abnormal trophoblast-endothelial replacement and later altered placenta (12).

Particularly, the VSMC remodeling of SA is a crucial process necessary to reduce arterial contractility, increase the luminal dilation, and transform spiral arterioles into thin-walled vessels with high capacitance and low resistance, thus to establish undisturbed blood flow toward the incipient placenta (Elia et al., 2011). In the process of smooth muscle wall remodeling, at mouse GD 7.5, undilated mesometrial distal decidual SA possesses a thick layer of VSMCs, but from GD 8–8.5, this muscular SA coat becomes loose and is almost undetectable in proximal decidua at GD 10.5–11 (Zhang et al., 2008; 2011). Therefore, recently, in mouse implantation site GD 10, we detected α-SMA-positive cells in SAs in the distal mesometrial decidua, but in the proximal one, near the trophoblastic layer, a negative reactivity for α-SMA in maternal vessels was observed (Ventureira et al., 2019).

During early pregnancy, both in humans and mice, decidual stromal cells and maternal leukocytes, located surrounding the vessels, acquire relevance in the regulation of maternal angiogenesis–vascularization and SA remodeling for preparation of maternal vessels via the expression of apoptotic molecules, endothelial cell mitogens, and proangiogenic and vasoactive factors (Whiteside et al., 2001; Das, 2009; 2010; Croy et al. 2011; Chambers et al., 2021). In the early mouse implantation site, decidual leukocytes comprise 30% of decidual stromal cells, and uNK cells constitute 70% of these leukocytes (Erlebacher 2013), while macrophages account for 20%. Regulatory T-cells and dendritic cells are also present in low numbers and are most involved in the immune tolerance of the semiallogeneic placenta and fetus.

In normal mouse pregnancy, precursors of uNK cells are found in the uterus from postnatal week 2 (Croy et al., 2009). They remain as small, agranular lymphocytes until blastocyst implantation and decidualization (GD 4.5) (Ashkar et al., 2003; Croy et al., 2003; 2006). There is a marked increase in uNK cells with the onset of decidualization (Sojka et al., 2019). Between GD 6-7, uNKs begin to proliferate rapidly and acquire cytoplasmic granules; however, at GD 7-7.5, the self-renewing uNK progenitor cells are also reported to be trafficked from primary and secondary lymphoid organs to the endometrium, where they rapidly proliferate (Chantakru et al., 2002). During normal early vascular remodeling, between GD 8.5 and GD 10.5, 10% of large, heavily granulated uNK cells are within lumens of decidual vessels, particularly small capillaries, about 25% of uNK cells are embedded within arterial walls, and the remainder associate with decidual stromal cells (van den Heuvel et al., 2005; Zhang et al., 2011; Ventureira et al., 2019). At the mouse mid-gestation period, uNKs achieve peak numbers and then gradually decline in number (Zhang et al., 2009). Thus, in mice, uNKs initiate SA remodeling (Kulandavelu et al. 2012), similarly as in human pregnancy (Robson et al., 2012), by decreasing the arterial wall SA-thickness and increasing vessel lumen diameter (Chakraborty et al., 2011; Harris et al., 2019; Sojka et al., 2019).

Uterine NKs promote angiogenesis by the expression of IFN-γ (Zang et al., 2011; Harris et al., 2019; Sojka et al., 2019), placental growth factor (PLGF), VEGF (Zang et al., 2011), TGFβ1, MMPs (Naruse et al., 2009), TGFβ-1, inducible nitric oxide synthase (iNOS) (Wang et al., 2003; Croy et al., 2006; Tayade et al., 2007), type 1 and type 2 receptors for angiotensin II, and NOS (Zang et al., 2011). Particularly, ECM remodeling and EC migration are attributed to MMP-2 and MMP-9 from uNKs localized around maternal blood vessels (Lima et al., 2014).

In pregnancy diseases and complications, failures in SA remodeling are associated with altered uNK cell number, activation, and signaling, which lead to decidual thickening, low maternal vascularization, and undilated hypertrophied spiral arteries with smooth muscle coats (Ashkar et al., 2000; Ventureira et al., 2019). Implantation sites deficient in uNK cells have anomalous features, diminished pruning of vessels, and poor development of lateral decidual sinuses (Lima et al., 2014). In the absence of uNK cells, the onset and progression of angiogenesis are delayed, leading to decreased oxygen tension, a hypoxia state that affects the trophoblast differentiation/invasive phenotype in mice (Chakraborty et al., 2011).

Decidual macrophages constitute about 10% of total uterine cells in the mouse and account for about 20%–25% of the leukocytes, a cell number that remains steady throughout the whole gestation (Chambers et al., 2021). They originate in immature monocytes released from the bone marrow into the bloodstream and are recruited to antimesometrial decidua by chemokines after circulating in the blood, where they differentiate into macrophages.

Macrophages are generally categorized into two subsets of polarization phenotypes, according to their cell surface markers and Th1/Th2 immune response of proinflammation or anti-inflammation properties: M1 macrophages, with proinflammatory responses, participate in apoptotic cell clearance and tissue remodeling (Sun et al., 2021), and M2 macrophages, with anti-inflammatory responses, sustain immune homeostasis and immunosuppression at the maternal–fetal interface (Nagamatsu and Schust, 2010). Differential metabolism of the amino acid arginine might also contribute to M1/M2 classification. Human and mouse decidual M1 macrophages are the products of the iNOS pathway in which arginine is converted to citrulline and nitric oxide (NO), enhancing cytotoxicity (Kong et al., 2015; Li et al., 2017), whereas M2 macrophages utilize the arginase pathway for the hydrolysis of arginine into urea and ornithine, which is important for cell proliferation and tissue repair (Neri et al., 1996). During peri-implantation, macrophages polarize into M1 macrophages to prepare for pregnancy, in response to chemokines secreted by decidual stromal cells (Yao et al., 2019), but a shift toward a more immune tolerant environment must occur for the pregnancy to continue, and increase of M2 begins (Ning et al., 2016). This change produces a mixed profile of M1/M2 macrophages that remains until mid-pregnancy, when the trophoblast invasion occurs in order to prevent fetal rejection (Jaiswal et al., 2012). Thereafter, a new shift toward a predominantly M2 phenotype occurs for extensive remodeling of the uterine vasculature (Hunt et al., 1984). Thus, both decidual proinflammatory and anti-inflammatory macrophage subsets contribute to the inflammatory balance during early gestation to maintain immune homeostasis at the maternal–fetal interface.

During the trophoblast-independent phase, decidual macrophages are clustered around spiral arteries at the site of implantation, highlighting their roles in decidual spiral artery angiogenesis, including ECM and SA remodeling. In humans, it was suggested that apoptosis of VSMCs within the remodeling vascular wall did not occur *in situ.* Instead of inducing VSMC apoptosis, macrophages mainly participate in phagocytosing apoptotic VSMCs that migrated into the surrounding stroma, where apoptosis occurs. As the disruption of VSMCs increases, the denser infiltration of decidual macrophages can be detected (Yao et al., 2019; Chambers et al., 2021; Sun et al., 2021).

Macrophages upregulate the expression of proangiogenic genes associated with extracellular matrix remodeling and endothelial proliferation, migration, tube formation, lumen, and branching (Yao et al., 2019). Decidual macrophages secrete the VEGF, placental growth factor (PlGF), and their receptors fms-like tyrosine kinase (Flt-1) (Yao et al., 2019), several cytokines, and MMPs, especially MMP-1, -2, -7, -9, and -10 (Choudhury et al., 2019; Harris et al., 2019). MMP-2 and -9 can alter chemokine bioactivity and induce leukocyte migration, and MMP-7 and -9 are critical in endometrial remodeling and enhancing trophoblast invasion (Chambers et al., 2021; Sun et al., 2021).

Altered maternal environment, oxidative stress, and cytokines can change the balance of M1/M2-type decidual macrophages and induce the secretion of anti-angiogenic factors (Li et al., 2017).

Overall, in healthy pregnancies, uNKs and macrophages are balanced toward an immunoregulatory phenotype, leading to adequate SA remodeling. Abnormal decidual macrophage number and lower levels of uNKs have been associated with impaired placental growth and malfunctioning in humans (Eide et al., 2006), IUGR (Williams et al., 2009), and pregnancy complications (Bezemer et al., 2020).

### Second trophoblast-dependent phase of maternal vascularization

The trophoblast-dependent phase is characterized by further VSMC separation and migration, loss of endothelial cells, fibrinoid deposition, and appearance of trophoblasts in both maternal lumen and vessels. At mouse mid-gestation, two main processes of decidual tissue and SA remodeling and reorganization occur at the decidual–trophoblast interface, depending on trophoblast actions (Rusidzé et al., 2023). One process is the interstitial trophoblast invasion into the decidual stroma, and the other involves the endovascular trophoblast invasion of the spiral arteries (Cross et al., 2002; Croy et al., 2012).

In the mouse implantation site GD 10–10.5, once the chorioallantoic placenta is established with the labyrinthine fetal vascular formation (Watson and Cross, 2005; Hu and Cross, 2010; Rai and Cross, 2014; Woods et al., 2018), continuous differentiation of P-TGCs and spongiotrophoblast cells takes place at the junctional zone (Cowden et al., 2005; Maltepe et al., 2005; Takeda et al., 2006; Soares et al., 2017; Gualdoni et al., 2021). From around GD 10.5–11, new differentiated trophoblasts begin major remodeling of the maternal SA and decidual stroma by interstitial invasion (Hu and Cross, 2010) at the decidual–trophoblastic interface (Figures 1.C, 1 step 4). To modify the composition of the decidual ECM and induce the degradation of the SA-basal membrane, both invasive P-TGC and GlyT cells secrete several MMPs (Whiteside et al., 2001; Harris, 2010; Varberg and Soares, 2021), thus actioning on SA-modifications proper of maternal vascular remodeling depending on trophoblast cells. From around mouse GD 11–11.5, SAs become invaded endovascularly and remodeled by invasive and migratory junctional spiral artery-associated trophoblast giant cells (SpA-TGCs) (Rossant and Cross, 2001; Adamson et al., 2002). Thus, SpA-TGCs infiltrate, degrade the SA basement membrane, induce endothelial apoptosis, and replace maternal endothelial cells by secretion of high amounts of MMPs (Whiteside et al., 2001). These processes, which allow maternal lacunae to become limited by trophoblastic cells acquiring a pseudoendothelial phenotype, result in the distension of arterial blood flow arriving at the JZ (Rai and Cross, 2014). However, in the mouse mid-gestation period, since this trophoblast endovascular invasion of SA is normally shallow compared to the human one, uNKs and macrophages may also play a role in completed SA remodeling through their MMP and NOS expressions (Moffet-King 2002; Huhn et al., 2021).

### MMP—NO pathways in abnormal early deciduous vascularization after perigestational alcohol consumption in an experimental mouse model

Gestational alcohol consumption can produce vascular resistance, vasodilation, and reduced blood flow in the placenta (Burd and Hofer, 2008; Ramadoss and Magness, 2012a). However, at present, little evidence suggests that perigestational alcohol consumption (PAC) up to early gestation induces abnormal placentation and leads to deficient growth and vasculopathy of the placenta at term. Reduced growth and malformed embryos (Gualdoni et al., 2021) or abnormal fetuses at term (Gualdoni et al., 2022a), induced by perigestational alcohol exposure up to organogenesis, were associated with altered placental indexes. Some studies in animal models explain the PAC-induced effects on the placenta by alterations in trophoblastic development and functionality (Gårdebjer et al., 2014; Kalisch-Smith and Moritz, 2017; Kalisch-Smith et al., 2019). Also, we recently showed, in a mouse model, that PAC up to peri-implantation or organogenesis disrupts the development of trophoblasts and induces deficient vascularization of the labyrinth (Perez-Tito et al., 2014; Gualdoni et al., 2021), leading to abnormal placenta later (Gualdoni et al., 2022a). However, early alterations in decidual angiogenesis during the trophoblast-independent phase may contribute primarily to insufficient maternal vascular development in the placenta at term. Here, we propose possible early sequential disrupted maternal angiogenic mechanisms induced by PAC leading to altered maternal vascularization in the definitive placenta.

During early gestation, mainly uNKs and decidual M2 macrophages regulate angiogenesis and remodeling of spiral arteries by expression/activity of the VEGF–MMP–NOS system. PAC up to early gestation (GD 7–10) can directly impact SA development, disrupting angiogenesis from the endometrium toward mesometrial decidual tissues in the implantation sites (Figures 2B, D, F vs Figures 2A, C, E). By producing oxidative stress (OS) (Coll et al., 2018) (Figure 2, Step 1), PAC reduces the uNK cell number in the mesometrial decidua (Ventureira et al., 2019) (Figure 2, Step 2), together with their VEGF production (Figure 2, Step 3). However, we recently showed that PAC also leads to reduced endothelial KDR expression (Ventureira et al., 2019), and in addition, activation of KDR (phosphorylated KDR) is increased, by which eNOS expression can be induced in CE and decidual tissue (Figure 2, Step 4). From alcohol-induced eNOS activation, high levels of NO are generated that, with reactive oxygen species, form peroxynitrites, contributing to OS increase (Coll et al., 2018) (Figure 2, Step 5). FLT-1 drives angiogenic modulation by its binding to VEGF (Lima et al., 2014), but its increase was associated with oxidative factors and turned into anti-angiogenic effects. PAC-decidual oxidative stress may induce increased FLT-1 expression (Ventureira et al., 2019) that, together with OS, activates KDR, thus contributing to VEGF-KDR expression disruption. Indeed, eNOS expression could also be triggered by FLT-1 (Bussolati et al., 2001) or induced by hypoxia since eNOS promoter contains hypoxia response elements (Schäffer et al., 2006). In this context, NO bioavailability decreases in maternal vasculature (Figure 2, Step 6), leading, in part, to reduced VSMC, EC, and basement membrane remodeling in maternal vessels of PAC-treated mice (Figure 2D, C). On the other hand, MMPs, derived from uNKs and/or other decidual stromal cells, may be altered in maternal tissue (Figure 2, Step 8), therefore worsening the deficient maternal vascular remodeling.

PAC-induced OS can be involved in changing the macrophage phenotype to a proinflammatory (M1) phenotype (Figure 2, Step 7), similar to the preeclamptic placenta in which a decreased M1/M2 macrophagic balance was observed in concordance with increased proinflammatory cytokines (Yao et al., 2019). Moreover, alcohol and OS may induce an inflammatory state in the decidua through an increase of lymphocytes (Figure 2, Step 8) and high levels of proinflammatory cytokines (Figure 2, Step 9) that impact positively to increase M1 macrophages (Figure 2, Step 7). This decidual OS inflammation can induce M1 macrophagic iNOS (Figure 2, Step 10) to produce high levels of NO (Figure 2, Step 11), which results in enhanced OS and apoptosis of cells and the ECM components of decidual tissue (Coll et al., 2018). In this maternal-altered environment, M1 macrophages probably secrete defective MMP levels, whose activation in the ECM could also be affected by OS–protease nitrosylation leading to loss of function and inhibition (Figure 2, Step 8). After PAC during early gestation, these VEGF–NO–MMP and OS-associated disrupted pathways may contribute to ECM and CE remodeling deficiencies by which the exposed spiral arteries have incomplete VSMC remodeling, EC detachment in the lumen, reduced CE proliferation, diminished lumen expansion, and vasodilation (Figure 2F, E). These mechanisms could explain the abnormal maternal vessels at the lateral sides of proximal mesometrial decidua, recently observed by us after PAC at GD 10 (Ventureira et al., 2019).

The immediate consequence of PAC-induced reduced dilation and less branching of maternal spiral arteries may be low blood perfusion and reduced oxygenation consistent with a persistent hypoxic–oxidative state at the junctional zone. High HIF-1 yields altered trophoblast growth, differentiation, and invasion (Gualdoni et al., 2021) that, together with earlier under-remodeled maternal SA, may lead to a deficient maternal EC remodeling/replacement from mid-gestation and subsequent abnormal definitive placenta (Figure 2, Step 12).

## Conclusion

In this review, we focused on and summarized the relevant process of early maternal vascularization for placental development and the roles of MMPs and NO as regulators of maternal angiogenesis in normal and abnormal placental conditions in early mouse pregnancy. We provide knowledge to understand better the importance of the research approach of these complex networks of molecular and cellular angiogenic mechanisms affected by maternal exposure to alcohol up to the early stages of pregnancy. Although it is well known that maternal alcohol ingestion is a significant risk factor for placentopathy induction, its association with fetal defects and, potentially, FASD is unclear. At present, few mouse models for studying the gestational and/or perigestational effects of alcohol consumption in placentation are available. In an experimental mouse model, here, we presented hypothetical mechanisms of alterations in early maternal vascularization–angiogenesis produced by perigestational administration of alcohol up to early gestation. This could be useful to explain, in part, the effects of maternal alcohol consumption until the first 2 months of human pregnancy on the feto-placental development at term.

Early failures of SA remodeling and primary maternal mechanisms of angiogenesis–vascularization due to PAC up to early stages of gestation can cause placental pathogenesis, which may underlie pregnancy complications, IUGR, FASD, and/or programming postnatal diseases. Alterations in MMP and NOS/NO mediators may modify placental functions, leading to pathological conditions of pregnancy. In this regard, based on our experience and literature exploration, few studies have described the maternal angiogenic-remodeling events at each stage of early development of the mouse implantation site. Bearing in mind that the murine model of placental development is not totally equal to the development of the human placenta, their similarities mean that a slightly more detailed description of early maternal angiogenesis–vascularization in the mouse model constitutes a contribution to the understanding of various similar physiopathological processes of angiogenesis and cellular and molecular mechanisms that are executed during vascular formation of the definitive placenta. In this sense, the goal of this review is to provide a theoretical–conceptual tool in the mouse experimental model for the analysis of the roles of decidual stromal and leukocyte cells and some angiogenic relevant molecules, such as MMPs and NO, in normal or altered vascularization during early pregnancy under alcohol exposure. However, there is a need to increase the knowledge of mouse experimental models that recapitulate the human gestation to better understand the etiology and pathogenesis of abnormal processes related to human placentopathies and their implications on fetal development and growth, produced by maternal alcohol consumption, at least, up to early gestation.

Studies on relevant angiogenic systems, such as the VEGF/R, MMPs, and NOS/NO, among others, in animal models should be continued, highlighting the importance of analyzing changes in angiogenic–vasoactive molecules in the normal placentation and their roles in altered pathways as etiological mechanisms of placental defects and risk for abnormal fetal growth due to maternal alcohol consumption.

## References

[B1] Aban C. Leguizamon G. F. Cella M. Damiano A. Franchi A. M. Farina M. G. (2013). Differential expression of endocannabinoid system in normal and preeclamptic placentas: effects on nitric oxide synthesis. Placenta 34, 67–74. 10.1016/j.placenta.2012.10.009 23122699

[B2] Adams R. H. Alitalo K. (2007). Molecular regulation of angiogenesis and lymphangiogenesis. Nat. Rev. Mol. Cell. Biol. 8, 464–478. 10.1038/nrm2183 17522591

[B3] Adamson S. Lu Y. Whiteley K. J. Holmyard D. Hemberger M. Pfarrer C. (2002). Interactions between trophoblast cells and the maternal and fetal circulation in the mouse placenta. Dev. Biol. 250, 358–373. 10.1016/s0012-1606(02)90773-6 12376109

[B4] Alexander C. M. Hansell E. J. Behrendtsen O. Flannery M. L. Kishnani N. S. Hawkes S. P. (1996). Expression and function of matrix metalloproteinases and their inhibitors at the maternal-embryonic boundary during mouse embryo implantation. Development 122, 1723–1736. 10.1242/dev.122.6.1723 8674412

[B5] Aliyu M. H. Lynch O. Nana P. N. Alio A. P. Wilson R. E. Marty P. J. (2011). Alcohol consumption during pregnancy and risk of placental abruption and placenta previa. *Matern. Child. Health*. J. 15, 670–676. 10.1007/s10995-010-0615-6 20437196

[B6] Aliyu M. H. Wilson R. E. Zoorob R. Chakrabarty S. Alio A. Kirby R. S. (2008). Alcohol consumption during pregnancy and the risk of early stillbirth among singletons. Alcohol 42, 369–374. 10.1016/j.alcohol.2008.04.003 18562153

[B7] Amălinei C. Căruntu I. D. Bălan R. A. (2007). Biology of metalloproteinases. Rom. J. Morphol. Embryol. 48 (4), 323–334. 18060181

[B8] Apte R. S. Chen D. S. Ferrara N. (2019). VEGF in signaling and disease: beyond discovery and development. Cell. 176, 1248–1264. 10.1016/j.cell.2019.01.021 30849371 PMC6410740

[B9] Ashkar A. A. Black G. P. Wei Q. He H. Liang L. Head J. R. (2003). Assessment of requirements for IL-15 and IFN regulatory factors in uterine NK cell differentiation and function during pregnancy. J. Immunol. 171, 2937–2944. 10.4049/jimmunol.171.6.2937 12960317

[B10] Ashkar A. A. Di Santo J. P. Croy B. A. (2000). Interferon gamma contributes to initiation of uterine vascular modification, decidual integrity, and uterine natural killer cell maturation during normal murine pregnancy. J. Exp. Med. 192, 259–270. 10.1084/jem.192.2.259 10899912 PMC2193246

[B11] Avalos L. A. Roberts S. C. M. Kaskutas L. A. Block G. Li D. K. (2014). Volume and type of alcohol during early pregnancy and the risk of miscarriage. Subst. Use. Misuse. 49, 1437–1445. 10.3109/10826084.2014.912228 24810392 PMC4183196

[B12] Bada H. S. Das A. Bauer C. R. Shankaran S. Lester B. M. Gard C. C. (2005). Low birth weight and preterm births: etiologic fraction attributable to prenatal drug exposure. J. Perinatol. 25, 631–637. 10.1038/sj.jp.7211378 16107872

[B13] Bany B. M. Harvey M. B. Schultz G. A. (2000). Expression of matrix metalloproteinases 2 and 9 in the mouse uterus during implantation and oil-induced decidualization. J. Reproduction Fertil. 120, 125–134. 10.1530/jrf.0.1200125 11006154

[B14] Bautch V. L. (2012). VEGF-directed blood vessel patterning: from cells to organism. Cold Spring Harb. Perspect. Med. 2, a006452. 10.1101/cshperspect.a006452 22951440 PMC3426816

[B15] Bezemer R. E. Schoots M. H. Timmer A. Scherjon S. A. Erwich J. J. H. M. van Goor H. (2020). Altered levels of decidual immune cell subsets in fetal growth restriction, stillbirth, and placental pathology. Front. Immunol. 11, 1898. 10.3389/fimmu.2020.01898 32973787 PMC7468421

[B16] Blois S. Klapp B. Barrientos G. (2011). Decidualization and angiogenesis in early pregnancy: unravelling the functions of DC and NK cells. J. Reproductive Immunol. 88, 86–92. 10.1016/j.jri.2010.11.002 21227511

[B17] Boeldt D. S. Yi F. X. Birde I. M. (2011). eNOS activation and NO function: pregnancy adaptive programming of capacitative entry responses alters nitric oxide (NO) output in vascular endothelium--new insights into eNOS regulation through adaptive cell signaling. J. Endocrinol. 210, 243–258. 10.1530/JOE-11-0053 21555345 PMC4059042

[B18] Brosens I. Puttemans P. Benagiano G. (2019). Placental bed research: i. The placental bed: from spiral arteries remodeling to the great obstetrical syndromes. Am. J. Obstet. Gynecol. 221, 437–456. 10.1016/j.ajog.2019.05.044 31163132

[B19] Bryan B. A. D’Amore P. A. (2007). What tangled webs they weave: rho-GTPase control of angiogenesis. Cell. Mol. Life Sci. 64, 2053–2065. 10.1007/s00018-007-7008-z 17530172 PMC11138424

[B20] Burd L. Hofer R. (2008). Biomarkers for detection of prenatal alcohol exposure: a critical review of fatty acid ethyl esters in meconium. Birth Defects Res. A Clin. Mol. Teratol. 82, 487–493. 10.1002/bdra.20464 18435469

[B21] Burd L. Roberts D. Olson M. Odendaal H. J. (2007). Ethanol and the placenta: a review. J. Matern. Fetal Neonatal Med. 20, 361–375. 10.1080/14767050701298365 17674239

[B22] Burton G. Woods A. Jauniaux E. Kingdom J. (2009). Rheological and physiological consequences of conversion of the maternal spiral arteries for uteroplacental blood flow during human pregnancy. Placenta 30, 473–482. 10.1016/j.placenta.2009.02.009 19375795 PMC2697319

[B23] Bussolati B. Dunk C. Grohman M. Kontos Ch.D. Ahmed J. M. Ahmed A. (2001). Vascular endothelial growth factor receptor-1 modulates vascular endothelial growth factor-mediated angiogenesis via nitric oxide. Am. J. Pathol. 159, 993–1008. 10.1016/S0002-9440(10)61775-0 11549592 PMC1850457

[B24] Carbone K. Pinto N. M. Abrahamsohn P. A. Zorn T. M. T. (2006). Arrangement and fine structure of collagen fibrils in the decidualized mouse endometrium. Microsc. Res. Tech. 69, 36–45. 10.1002/jemt.20265 16416410

[B25] Carter R. C. Wainwright H. Molteno C. D. Georgieff M. K. Dodge N. C. Warton F. (2016). Alcohol, methamphetamine, and marijuana exposure have distinct effects on the human placenta. Alcohol. Clin. Exp. Res. 40 (4), 753–764. 10.1111/acer.13022 27038593

[B26] Cebral E. Abrevaya X. C. Mudry M. D. (2011). Male and female reproductive toxicity induced by sub-chronic ethanol exposure in CF-1 mice. Cell. Biol. Toxicol. 27 (4), 237–248. 10.1007/s10565-011-9185-7 21331482

[B27] Cebral E. Faletti A. Jawerbaum A. Paz D. (2007). Periconceptional alcohol consumption-induced changes in embryonic prostaglandin E levels in mouse organogenesis. Modulation by nitric oxide. Prostagl. Leukot. Essent. Fat. Acids. 76, 141–151. 10.1016/j.plefa.2006.12.001 17276049

[B28] Chakraborty D. Karim Rumi M. A. Konno T. Soares M. J. (2011). Natural killer cells direct hemochorial placentation by regulating hypoxia-inducible factor dependent trophoblast lineage decisions. PNAS 108 (39), 16295–16300. 10.1073/pnas.1109478108 21900602 PMC3182743

[B29] Chambers M. Rees A. Cronin J. G. Nair M. Jones N. Thornton C. A. (2021). Macrophage plasticity in reproduction and environmental influences on their function. Front. Immunol. 11, 607328. 10.3389/fimmu.2020.607328 33519817 PMC7840613

[B30] Chantakru S. Miller C. Roach L. E. Kuziel W. A. Maeda N. Wang W. C. (2002). Contributions from self-renewal and trafficking to the uterine NK cell population of early pregnancy. J. Immunol. 168, 22–28. 10.4049/jimmunol.168.1.22 11751942

[B31] Chen J. Khalil R. A. (2017). Matrix metalloproteinases in normal pregnancy and preeclampsia. Prog. Mol. Biol. Transl. Sci. Vol. 148, 87–165. Chapter four. 10.1016/bs.pmbts.2017.04.001 PMC5548443 28662830

[B32] Chen L. He H. Cui N. Ren Z. Zhu M. Khalil R. A. (2020). Decreased uterine vascularization and uterine arterial expansive remodeling with reduced matrix metalloproteinase-2 and -9 in hypertensive pregnancy. Am. J. Physiol. Heart Circ. Physiol. 318, H165–H180. 10.1152/ajpheart.00602.2019 31834839 PMC6985805

[B33] Choudhury R. H. Dunk C. E. Lye S. J. HarrisAplin L. K. J. D, Jones R. L . (2019). Decidual leucocytes infiltrating human spiral arterioles are rich source of matrix metalloproteinases and degrade extracellular matrix *in vitro* and *in situ* . *Am. J. Reprod. Immuno*l. 81 (1), e13054. 10.1111/aji.13054 30267451

[B34] Chung A. S. Ferrara N. (2011). Developmental and pathological angiogenesis. Ann. Rev. Cell. Dev. Biol. 27, 563–584. 10.1146/annurev-cellbio-092910-154002 21756109

[B35] Coll T. A. Chaufan G. Pérez Tito L. G. Ventureira M. R. Ríos de Molina M. C. Cebral E. (2018). Cellular and molecular oxidative stress-related effects in uterine myometrial and trophoblast-decidual tissues after perigestational alcohol intake up to early mouse organogenesis. Mol. Cell. Biochem. 440 (1-2), 89–104. 10.1007/s11010-017-3158-y 28822072

[B36] Coll T. A. Chaufan G. Pérez-Tito L. Ventureira M. R. Sobarzo C. M. A. Ríos de Molina M. d. C. (2017). Oxidative stress and cellular and tissue damage in organogenic outbred mouse embryos after moderate perigestational alcohol intake. Mol. Reprod. Dev. 84, 1086–1099. 10.1002/mrd.22865 28708332

[B37] Coll T. A. Perez-Tito L. Sobarzo C. M. A. Cebral E. (2011). Embryo developmental disruption during organogenesis produced by CF-1 murine periconceptional alcohol consumption. Birth Defects Res. B 92, 560–574. 10.1002/bdrb.20329 21922637

[B38] Colvin L. Payne J. Parsons D. Kurinczuk J. J. Bower C. (2007). Alcohol consumption during pregnancy in nonindigenous west Australian women. Alcohol Clin. Exp. Res. 31, 276–284. 10.1111/j.1530-0277.2006.00303.x 17250620

[B39] Cowden Dahl K. D. Fryer B. H. Mack F. A. Compernolle V. Maltepe E. Adelman D. M. (2005). Hypoxia-inducible factors 1alpha and 2alpha regulate trophoblast differentiation. Mol. Cell. Biol. 25, 10479–10491. 10.1128/MCB.25.23.10479-10491.2005 16287860 PMC1291235

[B40] Creeth H. D. J. John R. M. (2020). The placental programming hypothesis: placental endocrine insufficiency and the co-occurrence of low birth weight and maternal mood disorders. Placenta 98, 52–59. 10.1016/j.placenta.2020.03.011 33039032

[B41] Cross J. Hemberger M. Lu Y. Nozaki T. Whiteley K. Masutani M. (2002). Trophoblast functions, angiogenesis and remodeling of the maternal vasculature in the placenta. Mol. Cell. Endocrinol. 187, 207–212. 10.1016/s0303-7207(01)00703-1 11988329

[B42] Croy B. A. Burke S. D. Barrette V. F. Zhang J. Hatta K. Smith G. N. (2011). Identification of the primary outcomes that result from deficient spiral arterial modification in pregnant mice. Pregnancy Hypertens. 1, 87–94. 10.1016/j.preghy.2010.10.002 22279618 PMC3264659

[B43] Croy B. A. Chen Z. Hofmann A. P. Lord E. M. Sedlacek A. Gerber S. A. (2012). Imaging of vascular development in early mouse decidua and its association with leukocytes and trophoblasts. Biol. Reprod. 87, 125. 10.1095/biolreprod.112.102830 22954796 PMC3509781

[B44] Croy B. A. Esadeg S. Chantakru S. van den Heuvel M. Paffaro V. A. He H. (2003). Update on pathways regulating the activation of uterine Natural Killer cells. Their interactions with decidual spiral arteries and homing of their precursors to the uterus. J. Reprod. Immunol. 59, 175–191. 10.1016/s0165-0378(03)00046-9 12896821

[B45] Croy B. A. van den Heuvel M. J. Borzychowski A. M. Tayade C. (2006). Uterine natural killer cells: a specialized differentiation regulated by ovarian hormones. Immunol. Rev. 214, 161–185. 10.1111/j.1600-065X.2006.00447.x 17100884

[B46] Croy B. A. Yamada A. De Mayo F. Adamson S. L. (2013). The guide to investigation of mouse pregnancy. 1st ed. Cambridge, MA, USA: Academic Press.

[B47] Croy B. A. Zhang J. Tayade C. Colucci F. Yadi H. Yamada A. T. (2009). Analysis of uterine natural killer cells in mice. Methods Mol. Biol. 612, 465–503. 10.1007/978-1-60761-362-6_31 20033660

[B48] Cui N. Hu M. Khalil R. A. (2017). Biochemical and biological attributes of matrix metalloproteinases. Prog. Mol. Biol. Transl. Sci. 147, 1–73. 10.1016/bs.pmbts.2017.02.005 28413025 PMC5430303

[B49] Das S. K. (2009). Cell cycle regulatory control for uterine stromal cell decidualization in implantation. Reproduction 137 (6), 889–899. 10.1530/REP-08-0539 19307426

[B50] Das S. K. (2010). Regional development of uterine decidualization: molecular signaling by hoxa-10. Mol. Reprod. Dev. 77 (5), 387–396. 10.1002/mrd.21133 19921737 PMC4267754

[B51] Davis-Anderson K. L. Berger S. Lunde-Young E. R. Naik V. D. Seo H. Johnson G. A. (2017). Placental proteomics reveal insights into fetal alcohol spectrum disorders. Alcohol. Clin. Exp. Res. 41, 1551–1558. 10.1111/acer.13448 28722160 PMC5581221

[B52] Dey S. K. Lim H. Das S. K. Reese J. Paria B. C. Daikoku T. (2004). Molecular cues to implantation. Endocr. Rev. 25, 341–373. 10.1210/er.2003-0020 15180948

[B53] Distler J. H. Hirth A. Kurowska-Stolarska M. Gay R. E. Gay S. Distler O. (2003). Angiogenic and angiostatic factors in the molecular control of angiogenesis. Q. J. Nucl. Med. 47 (3), 149–161. 12897707

[B54] Dubois B. Arnold B. Opdenakker G. (2000). Gelatinase B deficiency impairs reproduction. J. Clin. Investig. 106 (5), 627–628. 10.1172/JCI10910 10974013 PMC381291

[B55] Dubois B. Masure S. Hurtenbach U. Paemen L. Heremans H. van den Oord J. (1999). Resistance of young gelatinase B-deficient mice to experimental autoimmune encephalomyelitis and necrotizing tail lesions. J. Clin. Investig. 104 (11), 1507–1515. 10.1172/JCI6886 10587514 PMC409857

[B56] Eelen G. de Zeeuw P. Treps L. Harjes U. Wong B. W. Carmeliet P. (2017). Endothelial cell metabolism. Physiol. Rev. 98, 3–58. 10.1152/physrev.00001.2017 PMC5866357 29167330

[B57] Eide I. P. Rolfseng T. Isaksen C. V. Mecsei R. Roald B. Lydersen S. (2006). Serious foetal growth restriction is associated with reduced proportions of natural killer cells in decidua basalis. Virchows Arch. 448, 269–276. 10.1007/s00428-005-0107-z 16328353

[B58] Elia A. Charalambous F. Georgiades P. (2011). New phenotypic aspects of the decidual spiral artery wall during early post-implantation mouse pregnancy. Biochem. Biophysical Res. Commun. 416, 211–216. 10.1016/j.bbrc.2011.11.029 22100651

[B59] Erlebacher A. (2013). Immunology of the maternal–fetal interface. Annu. Rev. Immunol. 31, 387–411. 10.1146/annurev-immunol-032712-100003 23298207

[B60] Esper R. J. Nordaby R. A. Vilarino J. O. Paragano A. Cacharron J. L. Machado R. A. (2006). Endothelial dysfunction: a comprehensive appraisal. Cardiovasc. Diabetol. 5, 4. 10.1186/1475-2840-5-4 16504104 PMC1434727

[B61] Espino Y. Sosa S. Flores-Pliego A. Espejel-Nuñez A. Medina-Bastidas D. Vadillo-Ortega F. (2017). New insights into the role of matrix metalloproteinases in preeclampsia. Int. J. Mol. Sci. 18 (7), 1448. 10.3390/ijms18071448 28726716 PMC5535939

[B62] Fontana V. Coll T. A. Sobarzo C. M. A. Perez Tito L. Calvo J. C. Cebral E. (2012). Matrix metalloproteinase expression and activity in trophoblast-decidual tissues at organogenesis in CF-1 mouse. J. Mol. Histology 43 (5), 487–496. 10.1007/s10735-012-9429-8 22714107

[B63] Förstermann U. Sessa W. C. (2012). Nitric oxide synthases: regulation and function. Eur. Heart J. 33, 829–837. 10.1093/eurheartj/ehr304 21890489 PMC3345541

[B64] Gårdebjer E. M. Cuffe J. S. M. Pantaleon M. Wlodek M. E. Moritz K. M. (2014). Periconceptional alcohol consumption causes fetal growth restriction and increases glycogen accumulation in the late gestation rat placenta. Placenta 35, 50–57. 10.1016/j.placenta.2013.10.008 24239160

[B65] Grochot-Przeczek A. Florczyk U. Jozkowicz A. Dula J. Szade A. (2015). Cellular and molecular mechanisms of inflammation-induced angiogenesis. Int. Union Biochem. Mol. Biol. 67, 145–159. 10.1002/iub.1358 25899846

[B66] Gualdoni G. S. Gomez Castro G. Hernández R. Barbeito C. Cebral E. (2022c). Comparative matrix metalloproteinase-2 and -9 expression and activity during endotheliochorial and hemochorial trophoblastic invasiveness. Tissue Cell. 74, 101698. 10.1016/j.tice.2021.101698 34871824

[B67] Gualdoni G. S. Jacobo P. V. Barril C. Ventureira M. R. Cebral E. (2022b). Early abnormal placentation and evidence of vascular endothelial growth factor system dysregulation at the feto-maternal interface after periconceptional alcohol consumption. Front. Physiology (Front. Physiol) 12, 815760. 10.3389/fphys.2021.815760 PMC8847216 35185604

[B68] Gualdoni G. S. Pérez-Tito L. Barril C. Sobarzo C. Cebral E. (2022a). Abnormal growth and morphogenesis of placenta at term is linked to adverse fetal development after perigestational alcohol consumption up to early gestation in mouse. Birth Defects Res. 114, 611–630. 10.1002/bdr2.2063 35775613

[B69] Gualdoni G. S. Ventureira M. R. Coll T. A, Palomino W. A. Barbeito C. G. Cebral E. (2021). Perigestational alcohol consumption induces altered early placentation and organogenic embryo growth restriction by disruption of trophoblast angiogenic factors. Reprod. Biomed. Online, 42(3): 481–504. 10.1016/j.rbmo.2020.10.015 33549483

[B70] Gundogan F. Elwood G. Longato L. Tong M. Feijoo A. Carlson R. I. (2008). Impaired placentation in fetal alcohol syndrome. Placenta 29, 148–157. 10.1016/j.placenta.2007.10.002 18054075 PMC2692437

[B71] Gundogan F. Elwood G. Mark P. Feijoo A. Longato L. Tong M. (2010). Ethanol-induced oxidative stress and mitochondrial dysfunction in rat placenta: relevance to pregnancy loss. Alcohol. Clin. Exp. Res. 34, 415–423. 10.1111/j.1530-0277.2009.01106.x 20028358 PMC2952434

[B72] Gundogan F. Gilligan J. Qi W. Chen E. Naram R. de la Monte S. M. (2015). Dose effect of gestational ethanol exposure on placentation and fetal growth. Placenta 36, 523–530. 10.1016/j.placenta.2015.02.010 25745824 PMC4416060

[B73] Gupta K. K. Gupta V. K. Shirasaka T. (2016). An update on fetal alcohol syndrome—Pathogenesis, risks, and treatment. Alcohol. Clin. Exp. Res. 40, 1594–1602. 10.1111/acer.13135 27375266

[B74] Guttmacher A. E. Maddox Y. T. Spong C. Y. (2014). The human placenta project: placental structure, development, and function in real time. Placenta 35, 303–304. 10.1016/j.placenta.2014.02.012 24661567 PMC3999347

[B75] Gyselaers W. (2023). Origins of abnormal placentation: why maternal veins must not be forgotten. Am. J. Obstet. Gynecol. 228 (6), 613–621. 10.1016/j.ajog.2022.12.014 36539026

[B76] Haghighi Poodeh S. Salonurmi T. Nagy I. Koivunen P. Vuoristo J. Räsänen J. (2012). Alcohol-induced premature permeability in mouse placenta-yolk sac barriers *in vivo* . Placenta 33, 866–873. 10.1016/j.placenta.2012.07.008 22884851

[B77] Hamutoğlu R. Bulut H. E. Kaloğlu C. Önder O. Dağdeviren T. Aydemir M. N. (2020). The regulation of trophoblast invasion and decidual reaction by matrix metalloproteinase‐2, metalloproteinase‐7, and metalloproteinase‐9 expressions in the rat endometrium. Reprod. Med. Biol. 19 (4), 385–397. 10.1002/rmb2.12342 33071641 PMC7542015

[B78] Han M. Neves A. L. Serrano M. Brinez P. Huhta J. C. Acharya G. (2012). Effects of alcohol, lithium, and homocysteine on nonmuscle myosin-II in the mouse placenta and human trophoblasts. Am. J. Obstet. Gynecol. 207 (140), e7–e19. 10.1016/j.ajog.2012.05.007 PMC3408570 22704764

[B79] Hanson M. A. Gluckman P. D. (2008). Developmental origins of health and disease: new insights. Basic Clin. Pharmacol. Toxicol. 102, 90–93. 10.1111/j.1742-7843.2007.00186.x 18226060

[B80] Harris L. K. Benagiano M. D’Elios M. M. Brosens I. Benagiano G. (2019). Placental bed research: ll. Functional and immunological investigations of the placental bed. Am. J. Obstetrics Gynecol. 221 (5), 457–469. 10.1016/j.ajog.2019.07.010 31288009

[B81] Harris L. (2010). Review: trophoblast-Vascular cell interactions in early pregnancy: how to remodel a vessel. Placenta 31, S93–S98. 10.1016/j.placenta.2009.12.012 20060584

[B82] Hefler L. A. Reyes C. A. O’Brien W. E. Gregg A. R. (2001). Perinatal development of endothelial nitric oxide synthase-deficient mice. Biol. Reprod. 64 (2), 666–673. 10.1095/biolreprod64.2.666 11159371

[B83] Hellstrom M. Kun Phng L. Hofmann J. J. Wallgard E. Coultas L. Lindblom P. (2007). Dll4 signalling through Notch1 regulates formation of tip cells during angiogenesis. Nature 445 (7129), 776–780. 10.1038/nature05571 17259973

[B84] Henriet P. Emonard H. (2019). Matrix metalloproteinase-2: not (just) a “hero” of the past. Biochimie 166, 223–232. 10.1016/j.biochi.2019.07.019 31362036

[B85] Heo S. H. Choi Y. J. Ryoo H. M. Cho J. Y. (2010). Expression profiling of ETS and MMP factors in VEGF-activated endothelial cells: role of MMP-10 in VEGF-induced angiogenesis. J. Cell. Physiol. 224, 734–742. 10.1002/jcp.22175 20432469

[B86] Holbrook B. D. Davies S. Cano S. Shrestha S. Jantzie L. L. Rayburn W. F. (2019). The association between prenatal alcohol exposure and protein expression in human placenta. Birth Defects Res. 111, 749–759. 10.1002/bdr2.1488 30891944 PMC6946025

[B87] Hoyme H. E. Kalberg W. O. Elliott A. J. Blankenship J. Buckley D. Marais A. S. (2016). Updated clinical guidelines for diagnosing fetal alcohol spectrum disorders. Pediatrics 138, e20154256. 10.1542/peds.2015-4256 27464676 PMC4960726

[B88] Hu D. Cross J. (2010). Development and function of trophoblast giant cells in the rodent placenta. Inter. J. Dev. Biol. 54, 341–354. 10.1387/ijdb.082768dh 19876834

[B89] Huhn O. Zhao X. Esposito L. Moffett A. Colucci F. Sharkey A. M. (2021). How do uterine natural killer and innate lymphoid cells contribute to successful pregnancy? Front. Immunol. 12, 607669. 10.3389/fimmu.2021.607669 34234770 PMC8256162

[B90] Hulboy D. L. Rudolph L. A. Matrisian L. M. (1997). Matrix metalloproteinases as mediators of reproductive function. N. Mol. Hum. Reprod. 3 (1), 27–45. 10.1093/molehr/3.1.27 9239706

[B91] Hunt J. S. Manning L. S. Wood G. W. (1984). Macrophages in murine uterus are immunosuppressive. Cell. Immunol. 85, 499–510. 10.1016/0008-8749(84)90262-4 6232005

[B92] Iveli M. F. Morales S. Rebolledo A. Savietto V. Salemme S. Apezteguia M. (2007). Effects of light ethanol consumption during pregnancy: increased frequency of minor anomalies in the newborn and altered contractility of umbilical cord artery. Pediatr. Res. 61 (4), 456–461. 10.1203/pdr.0b013e3180332c59 17515871

[B93] Jacob-Ferreira A. L. Schulz R. (2013). Activation of intracellular matrix metalloproteinase-2 by reactive oxygen–nitrogen species: consequences and therapeutic strategies in the heart. Arch. Biochem. Biophys. 540, 82–93. 10.1016/j.abb.2013.09.019 24103691

[B94] Jaiswal M. K. Mallers T. M. Larsen B. Kwak-Kim J. Chaouat G. Gilman-Sachs A. (2012). V-ATPase upregulation during early pregnancy: a possible link to establishment of an inflammatory response during preimplantation period of pregnancy. Reproduction 143, 713–725. 10.1530/REP-12-0036 22454532

[B95] Kalisch-Smith J. I. Moritz K. M. (2017). Detrimental effects of alcohol exposure around conception: putative mechanisms. Biochem. Cell. Biol. 96, 107–116. 10.1139/bcb-2017-0133 29112458

[B96] Kalisch-Smith J. I. Steane S. E. Simmons D. G. Pantaleon M. Anderson S. T. Akison L. K. (2019). Periconceptional alcohol exposure causes female specific perturbations to trophoblast differentiation and placental formation in the rat. Development 146, dev172205. 10.1242/dev.172205 31182432

[B97] Khong T. Y. Mooney E. E. Ariel I. Balmus N. C. M. Boyd T. K. Brundler M. A. (2016). Sampling and definitions of placental lesions amsterdam placental workshop group consensus statement. Arch. Pathol. Lab. Med. 140, 698–713. 10.5858/arpa.2015-0225-CC 27223167

[B98] Kimura H. Esumi H. (2003). Reciprocal regulation between nitric oxide and vascular endothelial growth factor in angiogenesis. *Acta Biochim.* Pol. 50 (1), 49–59. 12673346

[B99] Kong L. Zhang Q. Chao J. Wen H. Zhang Y. Chen H. (2015). Polarization of macrophages induced by *Toxoplasma gondii* and its impact on abnormal pregnancy in rats. Acta Trop. 143, 1–7. 10.1016/j.actatropica.2014.12.001 25496968

[B100] Krause B. J. Hanson M. A. Casanello P. (2011). Role of nitric oxide in placental vascular development and function. Placenta 32(2011) 797–805. 10.1016/j.placenta.2011.06.025 21798594 PMC3218217

[B101] Kulandavelu S. Whiteley K. J. Qu D. Mu J. Bainbridge S. A. Adamson S. L. (2012). Endothelial nitric oxide synthase deficiency reduces uterine blood flow, spiral artery elongation, and placental oxygenation in pregnant mice. Hypertension 60, 231–238. 10.1161/HYPERTENSIONAHA.111.187559 22615111

[B102] Kwan S. T. C. Kezer C. A. Helfrich K. K. Saini N. Huebner S. M. Flentke G. R. (2020). Maternal iron nutriture modulates placental development in a rat model of fetal alcohol spectrum disorder. Alcohol 84, 57–66. 10.1016/j.alcohol.2019.11.003 31734307 PMC7131893

[B103] Lacko L. Hurtado R. Hinds S. Poulos M. Butler J. Stuhlmann H. (2017). Altered feto-placental vascularization, feto-placental malperfusion and fetal growth restriction in mice with Egfl7 loss of function. Development 144, 2469–2479. 10.1242/dev.147025 28526753 PMC5536866

[B104] Li H. Qu D. McDonald A. Isaac S. M. Whiteley K. J. Sung H. K. (2014). Trophoblast-specific reduction of VEGFA alters placental gene expression and maternal cardiovascular function in mice. Biol. Reprod. 91, 87. 10.1095/biolreprod.114.118299 25122061

[B105] Li Z. Zhao M. Li T. Zheng J. Liu X. Jiang Y. (2017). Decidual macrophage functional polarization during abnormal pregnancy due to toxoplasma gondii: role for LILRB4. Front. Immunol. 8, 1013. 10.3389/fimmu.2017.01013 28883820 PMC5573710

[B106] Lima P. D. Zhang J. Dunk C. Lye S. J. Croy B. A. (2014). Leukocyte driven-decidual angiogenesis in early pregnancy. Cell. Mol. Immunol. 11, 522–537. 10.1038/cmi.2014.63 25066422 PMC4220841

[B107] Linask K. K. Han M. (2016). Acute alcohol exposure during mouse gastrulation alters lipid metabolism in placental and heart development: rolate prevention. Birth Defects Res. A 106, 749–760. 10.1002/bdra.23526 PMC5094567 27296863

[B108] Lui S. Jones R. L. Robinson N. J. Greenwood S. L. Aplin J. D. Tower C. L. (2014). Detrimental effects of ethanol and its metabolite acetaldehyde, on first trimester human placental cell turnover and function. PLoS One 9, e87328. 10.1371/journal.pone.0087328 24503565 PMC3913587

[B109] Maltepe E. Krampitz G. W. Okazaki K. M. Red-Horse K. Mak W. Simon M. C. (2005). Hypoxia-inducible factor-dependent histone deacetylase activity determines stem cell fate in the placenta. Development 132, 3393–3403. 10.1242/dev.01923 15987772

[B110] Mandala M. Osol G. (2011). Physiological remodelling of the maternal uterine circulation during pregnancy. Basic Clin. Pharmacol. Toxicol. 110, 12–18. 10.1111/j.1742-7843.2011.00793.x 21902814

[B111] Martín-Estal I. Rodriguez-Zambrano M. A. Castilla-Cortázar I. (2019). “Biochemical assessment of placental function,” in Fetal growth restrict (Cham: Springer), 83–116.

[B112] McMillen I. C. Robinson J. S. (2005). Developmental origins ofthe metabolic syndrome: prediction, plasticity, and programming. Physiol. Rev. 85, 571–633. 10.1152/physrev.00053.2003 15788706

[B113] Meyer-Leu Y. Lemola S. Daeppen J. B. Deriaz O. Gerber S. (2011). Association of moderate alcohol use and binge drinking during pregnancy with neonatal health. Alcohol. Clin. Exp. Res. 35, 1669–1677. 10.1111/j.1530-0277.2011.01513.x 21554334

[B114] Moffet-King A. (2002). Natural killer cells and pregnancy. Nat. Rev. Immunol. 2, 656–663. 10.1038/nri886 12209134

[B115] Moncada S. Higgs E. (2006). The discovery of nitric oxide and its role in vascular biology. Br. J. Pharmacol. 147 (1), S193–S201. 10.1038/sj.bjp.0706458 16402104 PMC1760731

[B116] Mu J. Adamson S. L. (2006). Developmental changes in hemodynamics of uterine artery, utero- and umbilicoplacental, and vitelline circulations in mouse throughout gestation. Am. J. Physiol. Heart Circ. Physiol. 291, H1421–H1428. 10.1152/ajpheart.00031.2006 16603699

[B117] Nagamatsu T. Schust D. J. (2010). The immunomodulatory roles of macrophages at the maternal-fetal interface. Reprod. Sci. 17 (3), 209–218. 10.1177/1933719109349962 20065301

[B118] Naruse K. Lash G. E. Innes B. A. Otun H. A. Searle R. F. Robson S. C. (2009). Localization of matrix metalloproteinase (MMP)-2, MMP-9 and tissue inhibitors for MMPs (TIMPs) in uterine natural killer cells in early human pregnancy. Hum. Reprod. 24 (3), 553–561. 10.1093/humrep/den408 19088110

[B119] Neri I. Mazza V. Galassi M. C. Volpe A. Facchinetti F. (1996). Effects of l-arginine on utero-placental circulation in growth-retarded fetuses. Acta Obstet. Gynecol. Scand. 75, 208–212. 10.3109/00016349609047088 8607330

[B120] Ning F. Liu H. Lash G. E. (2016). The role of decidual macrophages during normal and pathological pregnancy. Am. J. Reprod. Immunol. 75, 298–309. 10.1111/aji.12477 26750089

[B121] Nuttall R. K. Sampieri C. L. Pennington C. J. Gill S. E. Schult G. A. Edwards D. R. (2004). Expression analysis of the entire MMP and TIMP gene families during mouse tissue development. FEBS Lett. 563, 129–134. 10.1016/S0014-5793(04)00281-9 15063736

[B122] Odendaal H. Wright C. Schubert P. Boyd T. K. Roberts D. J. Brink L. (2020). Associations of maternal smoking and drinking with fetal growth and placental abruption. Eur. J. Obstet. Gynecol. Reprod. Biol. 253, 95–102. 10.1016/j.ejogrb.2020.07.018 32862031

[B123] Ohira S. Motoki N. Shibazaki T. Misawa Y. Inaba Y. Kanai M. (2019). Alcohol consumption during pregnancy and risk of placental abnormality: the Japan environment and children’s study. Sci. Rep. 9, 10259. 10.1038/s41598-019-46760-1 31312010 PMC6635355

[B124] Orzabal M. R. Lunde-Younga E. R. Ramireza J. I. Naika V. D. Hillhouseb A. Kongantib K. (2019). Gestational binge alcohol-induced alterations in maternal uterine artery transcriptome. Reprod. Toxicol. 87, 42–49. 10.1016/j.reprotox.2019.05.053 31078653 PMC6628922

[B125] Patra J. Bakker R. Irving H. Jaddoe V. W. Malini S. Rehm J. (2011). Dose-response relationship between alcohol consumption before and during pregnancy and the risks of low birthweight, preterm birth and small for gestational age (SGA)-a systematic review and meta-analyses. BJOG 118, 1411–1421. 10.1111/j.1471-0528.2011.03050.x 21729235 PMC3394156

[B126] Perez-Garcia V. Fineberg F. Wilson R. Murray A. Mazzeo C. I. Tudor C. (2018). Placentation defects are highly prevalent in embryonic lethal mouse mutants. Nature 555, 463–468. 10.1038/nature26002 29539633 PMC5866719

[B127] Perez-Tito L. G. Bevilacqua E. Cebral E. (2014). Peri-implantational *in vivo* and *in vitro* embryo trophoblast development after perigestational alcohol exposure in the CD-1 mouse. Drug. Chem. Toxicol. 37, 184–197. 10.3109/01480545.2013.834358 24116715

[B128] Plaks V. Rinkenberger J. Dai J. Flannery M. Sund M. Kanasaki K. (2013). Matrix metalloproteinase-9 deficiency phenocopies features of preeclampsia and intrauterine growth restriction. Proc. Natl. Acad. Sci. U. S. A. 110 (27), 11109–11114. 10.1073/pnas.1309561110 23776237 PMC3704020

[B129] Possomato-Vieira J. S. Khalil R. A. (2016). Mechanisms of endothelial dysfunction in hypertensive pregnancy and Preeclampsia. Adv. Pharmacol. 77, 361–431. 10.1016/bs.apha.2016.04.008 27451103 PMC4965238

[B130] Prefumo F. Sebire N. Thilaganathan B. (2004). Decreased endovascular trophoblast invasion in first trimester pregnancies with high-resistance uterine artery Doppler indices. Hum. Reprod. 19, 206–209. 10.1093/humrep/deh037 14688183

[B131] Qian J. Fulton D. (2013). Post-translational regulation of endothelial nitric oxide synthase in vascular endothelium. Front. Physiol. 4, 347. 10.3389/fphys.2013.00347 24379783 PMC3861784

[B132] Qin X-Y. Shen H-H. Zhang X-Y. Zhang X. Feng X. Wang W-J. 2023. Hypoxia-mediated chemotaxis and residence of macrophage in decidua by secreting VEGFA and CCL2 during normal pregnancy. Reproduction 165 (5), 543–555. 10.1530/REP-22-0473 36809184

[B133] Quintero-Fabián S. Arreola R. Becerril-Villanueva E. Torres-Romero J. C. Arana- Argáez V. Lara-Riegos J. (2019). Role of matrix metalloproteinases in angiogenesis and cancer. Front. Oncol. 9, 1370. 10.3389/fonc.2019.01370 31921634 PMC6915110

[B134] Radek K. A. Matthies A. M. Burns A. L. Heinrich S. A. Kovacs A. J. DiPietro L. A. (2005). Acute ethanol exposure impairs angiogenesis and the proliferative phase of wound healing. Am. J. Physiol. Heart. Circ. Physiol. 289, H1084–H1090. 10.1152/ajpheart.00080.2005 15863463

[B135] Rai A. Cross J. C. (2014). Development of the hemochorial maternal vascular spaces in the placenta through endothelial and vasculogenic mimicry. Dev. Biol. 387, 131–141. 10.1016/j.ydbio.2014.01.015 24485853

[B136] Ramadoss J. Magness R. R. (2012c). Alcohol-induced alterations in maternal uterine endothelial proteome: a quantitative iTRAQ mass spectrometric approach. Reprod. Toxicol. 34 (4), 538–544. 10.1016/j.reprotox.2012.08.008 22960358 PMC3513571

[B137] Ramadoss J. Magness R. R. (2012b). Multiplexed digital quantification of binge-like alcohol-mediated alterations in maternal uterine angiogenic mRNA transcriptome. Physiol. Genomics. 44, 622–628. 10.1152/physiolgenomics.00009.2012 22535877 PMC3426435

[B138] Ramadoss J. Magness R. (2012). Vascular effects of maternal alcohol consumption. Am. J. Physiol. Heart Circ. Physiol. 303, H414–H421. 10.1152/ajpheart.00127.2012 22730388 PMC3423142

[B139] Ramathal C. Y. Bagchi I. C. Taylor R. N. Bagchi M. K. (2010). Endometrial decidualization: of mice and men. Semin. Reprod. Med. 28, 17–26. 10.1055/s-0029-1242989 20104425 PMC3095443

[B140] Rätsep M. T. Felker A. M. Kay V. R. Tolusso L. Hofmann A. P. Croy A. B. (2015). Uterine natural killer cells: supervisors of vasculature construction in early decidua basalis. Reproduction 149, 149 R91–R102. 10.1530/REP-14-0271 25342175

[B141] Reynolds L. P. Caton J. S. Redmer D. A. Grazul-Bilska A. T. Vonnahme K. A. Borowicz P. P. (2006). Evidence for altered placental blood flow and vascularity in compromised pregnancies. J. Physiol. 572, 51–58. 10.1113/jphysiol.2005.104430 16469783 PMC1779650

[B142] Roberts J. M. Escudero C. (2012). The placenta in preeclampsia. Pregnancy Hypertens. 2, 72–83. 10.1016/j.preghy.2012.01.001 22745921 PMC3381433

[B143] Robson A. Harris L. K. Innes B. A. Lash G. E. Aljunaidy M. M. Aplin J. D. (2012). Uterine natural killer cells initiate spiral artery remodeling in human pregnancy. FASEB J. 26, 4876–4885. 10.1096/fj.12-210310 22919072

[B144] Rosenberg M. J. Wolff C. R. El-Emawy A. Staples M. C. Perrone-Bizzozero N. I. Savage D. D. (2010). Effects of moderate drinking during pregnancy on placental gene expression. Alcohol 44, 673–690. 10.1016/j.alcohol.2009.10.002 20053520 PMC3654802

[B145] Rossant J. Cross J. C. (2001). Placental development: lessons from mouse mutants. Nat. Rev. Genet. 2, 538–548. 10.1038/35080570 11433360

[B146] Rundhaug J. E. (2005). Matrix metalloproteinases and angiogenesis. J. Cell. Mol. Med. 9 (2), 267–285. 10.1111/j.1582-4934.2005.tb00355.x 15963249 PMC6740080

[B147] Rusidzé M. Gargaros A. Fébrissy C. Dubucs C. Weyl A. Ousselin J. (2023). Estrogen actions in placental vascular morphogenesis and spiral artery remodeling: a comparative view between humans and mice. Cells 12, 620. 10.3390/cells12040620 36831287 PMC9954071

[B148] Salihu H. M. Kornosky J. L. Lynch O. N. Alio A. P. August E. M. Marty P. J. (2011). Impact of prenatal alcohol consumption on placenta-associated syndromes. Alcohol 45, 73–79. 10.1016/j.alcohol.2010.05.010 20598485

[B149] Schäffer L. Vogel J. Breymann C. Gassmann M. Marti H. H. (2006). Preserved placental oxygenation and development during severe systemic hypoxia. Am. J. Physiol. Regul. Integr. Comp. Physiol. 290, R844–R851. 10.1152/ajpregu.00237.2005 16195499

[B150] Sharma D. Shastri S. Farahbakhsh N. Sharma P. (2016). Intrauterine growth restriction - Part 1. J. Matern. Fetal. Neonatal. Med. 29, 3977–3987. 10.3109/14767058.2016.1152249 26856409

[B151] Silva J. F. Serakides R. (2016). Intrauterine trophoblast migration: a comparative view of humans and rodents. Cell. Adh Migr. 10 (1-2), 88–110. 10.1080/19336918.2015.11203971080/19336918.2015.1120397 26743330 PMC4853047

[B152] Simmons D. G. Fortier A. L. Cross J. C. (2007). Diverse subtypes and developmental origins of trophoblast giant cells in the mouse placenta. Dev. Biol. 304, 567–578. 10.1016/j.ydbio.2007.01.009 17289015

[B153] Smith S. D. Dunk C. E. Aplin J. D. Harris L. K. Jones R. L. (2009). Evidence for immune cell involvement in decidual spiral arteriole remodeling in early human pregnancy. Am. J. Pathol. 174, 1959–1971. 10.2353/ajpath.2009.080995 19349361 PMC2671283

[B154] Soares M. J. Iqbal K. Kozai K. (2017). Hypoxia and placental development. Birth Defects Res. 109, 1309–1329. 10.1002/bdr2.1135 29105383 PMC5743230

[B155] Sojka D. K. Yang L. Yokoyama W. M. (2019). Uterine natural killer cells. Front. Immunol. 10, 960. 10.3389/fimmu.2019.00960 31118936 PMC6504766

[B156] Staun-Ram E. Goldman S. Gabarin D. Shalev E. (2004). Expression and importance of matrix metalloproteinase 2 and 9 (MMP-2 and -9) in human trophoblast invasion. Reprod. Biol. Endocrinol. 2, 59–13. 10.1186/1477-7827-2-59 15294019 PMC516041

[B157] Subramanian K. Naik V. D. Sathishkumar M. S. K. Yallampalli C. Saade G. R. Hankins G. D. (2014). Chronic binge alcohol exposure during pregnancy impairs rat maternal uterine vascular function. Alcohol. Clin. Exp. Res. 38 (7), 1832–1838. 10.1111/acer.12431 24962648 PMC4107157

[B158] Sun F. Songcun W. Meirong D. (2021). Functional regulation of decidual macrophages during pregnancy. J. Reprod. Immunol. 143, 103264. 10.1016/j.jri.2020.103264 33360717

[B159] Szade A. Grochot-Przeczek A. Florczyk U. Jozkowicz A. Dulak J. (2015). Cellular and molecular mechanisms of inflammation-induced angiogenesis. IUBMB Life 67 (3), 145–159. 10.1002/iub.1358 25899846

[B160] Tai M. Piskorski A. Kao J. C. W. Hess L. A. de la Monte S. M. Gündogan F. (2017). Placental morphology in fetal alcohol spectrum disorders. Alcohol Alcohol 52 (2), 138–144. 10.1093/alcalc/agw088 28182213 PMC6248725

[B161] Takeda k. Ho V. C. Takeda H. Duan L. J. Nagy A. Fong G. H. (2006). Placental but not heart defects are associated with elevated hypoxia-inducible factor alpha levels in mice lacking prolyl hydroxylase domain protein 2. Mol. Cell. Biol. 26, 8336–8346. 10.1128/MCB.00425-06 16966370 PMC1636770

[B162] Tayade C. Hilchie D. He H. Fang Y. Moons L. Carmeliet P. (2007). Genetic deletion of placenta growth factor in mice alters uterine NK cells. J. Immunol. 178, 4267–4275. 10.4049/jimmunol.178.7.4267 17371983

[B163] Teesalu T. Masson R. G. Basset P. Blas F. Talarico D. (1999). Expression of matrix metalloproteinases during murine chorioallantoic placenta maturation. Dev. Dyn. 214, 248–258. 10.1002/(SICI)1097-0177(199903)214:3<248::AID-AJA8>3.0.CO;2-N 10090151

[B164] Timokhina E. Strizhakov A. Ibragimova S. Gitel E. Ignatko I. Belousova V. (2020). Matrix metalloproteinases MMP-2 and MMP-9 occupy a new role in severe preeclampsia. J. Pregnancy 2020, 8369645. 10.1155/2020/8369645 33381317 PMC7759403

[B165] Torry D. S. Leavenworth J. Chang M. Maheshwari V. Groesch K. Ball E. R. (2007). Angiogenesis in implantation. J. Assist. Reprod. Genet. 24, 303–315. 10.1007/s10815-007-9152-7 17616801 PMC3455012

[B166] Valdés G. Erices R. Chacón C. Corthorn J. (2008). Angiogenic, hyperpermeability and vasodilator network *in utero*-placental units along pregnancy in the Guinea-pig (*Cavia porcellus*). Reproductive Biol. Endocrinol. 6, 13. 10.1186/1477-7827-6-13 PMC2291058 18371207

[B167] Van den Heuvel M. J. Chantakru S. Xie X. Evans S. S. Tekpetey F. Mote P. A. (2005). Trafficking of circulating pro-NK cells to the decidualizing uterus; regulatory mechanisms in the mouse and human. Immunol. Investig. 34 (3), 273–293. 10.1081/imm-200064488 16136782 PMC3286484

[B168] Vanhoutte P. M. Zhao Y. Xu A. Leung S. W. (2016). Thirty years of saying NO: sources, fate, actions, and misfortunes of the endothelium-derived vasodilator mediator. Circ. Res. 119, 375–396. 10.1161/CIRCRESAHA.116.306531 27390338

[B169] Varberg K. M. Soares M. J. (2021). Paradigms for investigating invasive trophoblast cell development and contributions to uterine spiral artery remodeling. Placenta 113, 48–56. 10.1016/j.placenta.2021.04.012 33985793 PMC8440336

[B170] Ventureira M. R. Sobarzo C. Argandoña F. Palomino W. A. Barbeito C. Cebral C. (2019). Decidual vascularization during organogenesis after perigestational alcohol ingestion. Reproduction 158, 109–122. 10.1530/REP-18-0230 31042673

[B171] Waker C. A. Hwang A. E. Bowman-Gibson S. Chandiramani C. H. Linkous B. Stone M. L. (2023). Mouse models of preeclampsia with preexisting comorbidities. Front. Physiol. 14, 1137058. 10.3389/fphys.2023.1137058 37089425 PMC10117893

[B172] Wang C. Tanaka T. Nakamura H. Umesaki N. Hirai K. Ishiko O. (2003). Granulated metrial gland cells in the murine uterus: localization, kinetics, and the functional role in angiogenesis during pregnancy. Microsc. Res. Tech. 60, 420–429. 10.1002/jemt.10280 12567399

[B173] Wang H. Keiser J. A. (1998). Vascular endothelial growth factor upregulates the expression of matrix metalloproteinases in vascular smooth muscle cells: role of flt-1. Circ. Res. 83, 832–840. 10.1161/01.res.83.8.832 9776730

[B174] Wang X. Matsumoto H. Zhao X. Das S. K. Paria B. C. (2004). Embryonic signals direct the formation of tight junctional permeability barrier in the decidualizing stroma during embryo implantation. J. Cell. Sci. 117, 53–62. 10.1242/jcs.00826 14627626

[B175] Watson E. D. Cross J. C. (2005). Development of structures and transport functions in the mouse placenta. Physiology 20, 180–193. 10.1152/physiol.00001.2005 15888575

[B176] Whiteside E. J. Jackson M. M. Herington A. C. Edwards D. R. Harvey M. B. (2001). Matrix metalloproteinase-9 and tissue inhibitor of metalloproteinase-3 are key regulators of extracellular matrix degradation by mouse embryos. Biol. Reprod. 64, 1331–1337. 10.1095/biolreprod64.5.1331 11319136

[B177] Williams P. J. Bulmer J. N. Searle R. F. Innes B. A. Robson S. C. (2009). Altered decidual leucocyte populations in the placental bed in pre-eclampsia and foetal growth restriction: a comparison with late normal pregnancy. Reproduction 138, 177–184. 10.1530/REP-09-0007 19357130

[B178] Woods L. Perez-Garcia V. Hemberger M. (2018). Regulation of placental development and its impact on fetal growth-new insights from mouse models. Front. Endocr. 9, 570. 10.3389/fendo.2018.00570 PMC6170611 30319550

[B179] Yao Y. Xu X-H. Jin L. (2019). Macrophage polarization in physiological and pathological pregnancy. Front. Immunol. 10, 792. 10.3389/fimmu.2019.00792 31037072 PMC6476302

[B180] Zhang J. Chen Z. Smith G. N. Croy A. B. (2011). Natural killer cell-triggered vascular transformation: maternal care before birth? Cell. Mol. Immunol. 8, 1–11. 10.1038/cmi.2010.38 20711229 PMC3079746

[B181] Zhang J. H. Yamada A. T. Croy B. A. (2009). DBA-Lectin reactivity defines natural killer cells that have homed to mouse decidua. Placenta 30, 968–973. 10.1016/j.placenta.2009.08.011 19765824

[B182] Zhang S. Mesalam A. Joo M. D. Lee K. L. Hwang J. Y. Xu L. (2020). Matrix metalloproteinases improves trophoblast invasion and pregnancy potential in mice. Theriogenology 151, 144–150. 10.1016/j.theriogenology.2020.02.002 32344273

[B183] Zhang J. Dong H. Wang B. Zhu S. Croy B. A. Croy B. A. (2008). Dynamic changes occur in patterns of endometrial EFNB2/EPHB4 expression during the period of spiral arterial modification in mice. Biol. Reprod. 79, 450–458. 10.1095/biolreprod.108.067975 18463357

[B184] Zhu Y. Romitti P. A. Caspers Conway K. M. Shen D. H. Sun L. Browne M. L. (2015). Maternal periconceptional alcohol consumption and congenital heart defects. Birth Defects Res. A Clin. Mol. Teratol. 103, 617–629. 10.1002/bdra.23352 26118863 PMC7668305

